# Empowering multifaceted analysis of spatial transcriptomics data with RGAST

**DOI:** 10.1093/bib/bbag298

**Published:** 2026-06-16

**Authors:** Yuqiao Gong, Xin Yuan, Zhangsheng Yu

**Affiliations:** Department of Bioinformatics and Biostatistics, School of Life Sciences and Biotechnology, Shanghai Jiao Tong University, 800 Dongchuan Road, Minhang District, Shanghai, Shanghai, 200240, China; Department of Bioinformatics and Biostatistics, School of Life Sciences and Biotechnology, Shanghai Jiao Tong University, 800 Dongchuan Road, Minhang District, Shanghai, Shanghai, 200240, China; SJTU-Yale Joint Center for Biostatistics and Data Science Organization, Shanghai Jiao Tong University, 800 Dongchuan Road, Minhang District, Shanghai, Shanghai, 200240, China; Institute of Translational Medicine, Shanghai Jiao Tong University, 800 Dongchuan Road, Minhang District, Shanghai, Shanghai, 200240, China; National Center for Translational Medicine, Shanghai Jiao Tong University, 800 Dongchuan Road, Minhang District, Shanghai, Shanghai, 200240, China; Department of Bioinformatics and Biostatistics, School of Life Sciences and Biotechnology, Shanghai Jiao Tong University, 800 Dongchuan Road, Minhang District, Shanghai, Shanghai, 200240, China; SJTU-Yale Joint Center for Biostatistics and Data Science Organization, Shanghai Jiao Tong University, 800 Dongchuan Road, Minhang District, Shanghai, Shanghai, 200240, China; Institute of Translational Medicine, Shanghai Jiao Tong University, 800 Dongchuan Road, Minhang District, Shanghai, Shanghai, 200240, China; Institute of Clinical Medicine, School of Medicine, Shanghai Jiao Tong University, 800 Dongchuan Road, Minhang District, Shanghai, Shanghai, 200240, China

**Keywords:** spatial transcriptomics, heterogeneous graph network, relational graph attention, cell–cell communication, RGAST

## Abstract

Spatial transcriptomics (ST) enables mapping gene expression in native tissue context to resolve architecture and cellular interactions, but current analytical workflows rely on separate algorithms for distinct tasks. We present RGAST (Relational Graph Attention network for ST analysis), a framework that builds upon and extends our earlier HERGAST model (specifically designed for large-scale ST data analysis) for diverse downstream analysis. By introducing a relational graph attention auto-encoder, RGAST jointly models spatial proximity and gene expression similarity to capture both local and global structures in ST data. This design enables a wide range of downstream tasks within a single framework. Through comprehensive benchmarking, RGAST demonstrates superior performance in spatial domain identification across multiple platforms, improving adjusted rand index by ~10% compared to the second-best model in the dorsolateral prefrontal cortex dataset. RGAST accurately reconstructs known neuroglial interaction patterns in the mouse hypothalamus, including long-range signaling pathways that are often missed by distance-constrained methods. Moreover, RGAST also excels in boosting spatially variable gene identification accuracy, delivering more precise inference of developmental trajectories in the human cortex, and robust reconstruction of 3D tissue architectures from serial sections. Collectively, these results establish RGAST as a powerful tool for providing coherent solution to advance ST data analysis across multiple research scenarios.

## Introduction

Spatial transcriptomics (ST) technologies are revolutionizing biology by mapping gene expression in the native tissue context. The rapid evolution of these platforms, from commercial systems like 10× Visium [1] to high-resolution methods such as MERFISH [2] and Stereo-seq [3], has led to an unprecedented explosion in data scale and complexity. This has spurred the development of computational methods to perform critical downstream analyses, including identifying spatial domains, detecting spatially variable genes (SVGs), inferring developmental trajectories, and resolving cell–cell communication (CCC) networks.

While numerous computational tools have been developed, including integration toolkits like Seurat [4] and specialized algorithms like SEDR [5], conST [6], SpaGCN [7], BayesSpace [8], STAGATE [9], GraphST [10], DeepLinc [11], Scriabin [12], NICHES [13], and COMMOT [14], the analytical landscape remains fragmented. Most tools focus on a subset of tasks, creating analytical silos where tasks like spatial domain identification and cell–cell interaction inference are conducted as disconnected steps. Another issue lies on the limited global pattern recognition, as identifying complex phenomena like long-range, cross-regional cellular communication becomes difficult when the framework is not designed to holistically integrate information from the entire tissue slice. This makes it challenging to derive consistent, comprehensive insights from a single ST dataset. Our previously developed HERGAST model [15] made an advance by using a heterogeneous graph network to integrate spatial proximity and gene expression similarity, resulting in superior spatial clustering and signal amplification. However, its focus was narrow, and the full potential of this powerful architecture to serve as a foundation for the broad spectrum of ST analyses remained unexplored, leaving a critical gap in the field.

To fill this gap, we developed RGAST (Relational Graph Attention network for ST analysis), a comprehensive analytical framework that builds upon the core success of HERGAST but significantly expands its practical utility. While RGAST retains the powerful heterogeneous graph attention auto-encoder from HERGAST to generate robust latent embeddings, its key innovations lie in how it leverages the model’s outputs. RGAST introduces two major advancements. First, it goes beyond merely using the embeddings by further modeling the learned attention scores from the graph network. We leveraged these scores to develop an integrated module for *de novo* CCC analysis, enabling the direct inference of high-confidence interaction networks, including those acting over long distances. Second, RGAST is structured as a unified platform with a suite of compatible downstream analysis modules. These modules are designed to seamlessly utilize the high-quality embeddings to enhance a wide spectrum of tasks. This unified approach leads to more accurate spatial clustering, sensitive SVG detection, robust inference of developmental trajectories, and coherent reconstruction of 3D tissue architectures. By transforming a powerful core model into a multifunctional analytical toolkit, RGAST provides a more holistic and integrated solution for extracting deep biological insights from complex ST data.

In this work, we comprehensively demonstrate the superior performance and versatility of RGAST. We first establish its state-of-the-art (SOTA) performance in spatial domain identification across diverse ST platforms. We then showcase its application in inferring CCC networks by successfully validating known neural signaling pathways. Finally, we illustrate its utility in other critical analyses, including SVG detection, trajectory inference, and 3D tissue architecture reconstruction. We believe that RGAST, as a unified, efficient, and powerful framework, will significantly empower researchers to extract deeper biological insights from the growing complexity of spatial omics data.

## Materials and methods

### Datasets and preprocessing

We applied RGAST to ST datasets generated by different platforms, including MERFISH, HDST, 10× Visium, Stereo-seq, and seqFISH+. Specifically, the MERFISH dataset was a spatially resolved cell atlas of the mouse hypothalamic preoptic region, profiling ~1 million cells, each with only 155 genes [16]. We selected six consecutive slices from a single animal sample to conduct our experiments. After removing the putative doublets first, the number of spots ranged from 4787 to 5926 for each section. The HDST breast cancer dataset includes 3 tissue sections, which were obtained from a histological grade 3 HER2+ patient [17]. We selected a field of CN21_E2 (spot_x:550~700, spot_y:550~650) and removed all cells with unknown cell type or niche annotation. The 10× Visium DLPFC dataset included 12 human DLPFC sections sampled from 3 individuals. The number of spots ranged from 3498 to 4789 for each section, and the original authors manually annotated the areas of DLPFC layers and WM [18]. The human intestine dataset was a developmental system dataset from human fetal intestine samples collected at 12 and 19 post-conception weeks (PCWs) [19]. We used the A3 sample in our research, which contained only 434 spots. The Stereo-seq mouse olfactory bulb data was binned into a resolution of cellular levels (~14 μm) and contained 19 109 spots [5]. The MERFISH dataset was a spatially resolved cell atlas of the mouse hypothalamic preoptic region, profiling ~1 million cells, each with only 155 genes [16]. We selected six consecutive slices from a single animal sample to conduct our experiments. After preprocessing, the number of spots ranged from 4787 to 5926 for each section. The mouse cortex seqFISH+ dataset contained mRNAs for 10 000 genes in single cells, with high accuracy and sub-diffraction-limit resolution. This dataset had a spot number of 913 [20]. The data sources are summarized in Supplementary Table S1.

For all datasets, we normalized and scaled the data, then performed PCA using the Scanpy package [21]. The resulting principal components for each spot were used as input to RGAST.

### Construction of relational graph

The spatial neighborhood relationship is established by considering the Euclidean distance between the spatial locations of different spots. In the case of 10× Visium data, we set the graph to include the six nearest neighbors for each spot. For other datasets, we define the adjacency matrix, $A$, such that ${A}_{ij}$ = 1 if and only if the Euclidean distance between spot $i$ and spot $j$ is less than a predefined hyperparameter, $r$. The expression similarity relationship is constructed by considering the Euclidean distance of the PCA representation of the gene expression spectra of different spots.

### Relational graph attention auto-encoder

The relational graph attention auto-encoder consists of an encoder of two relational graph attention layer and a linear layer decoder.

#### Encoder

The input of the encoder in our architecture is a graph with $R=2$ relation types and $N$ nodes (spots). The ${i}_{th}$ node is represented by a feature vector of the PCA dimensional reduction representation of gene expressions ${\boldsymbol{x}}_i$. In our study, we use 200 PCs for all datasets except 100 PCs for the MERFISH data (which contains less than 200 genes). Query and key representations for the ${l}_{th}$ encoder layer are computed for each relation type with the help of both query and key kernels, i.e.


(1) \begin{eqnarray*} {q}_i^{(r)}={\boldsymbol{W}}_l^{(r)}{\boldsymbol{x}}_i\,\cdotp{\boldsymbol{Q}}^{(r)} \end{eqnarray*}

(2) \begin{eqnarray*} {k}_i^{(r)}={\boldsymbol{W}}_l^{(r)}{\boldsymbol{x}}_i\, \cdotp{\boldsymbol{K}}^{(r)} \end{eqnarray*}

where ${\boldsymbol{W}}_l$ is the trainable weight matrix of layer $l$. Then, additive attention [22] is applied to compute attention logits ${a}_{ij}^{(r)}$:


(3) \begin{eqnarray*} {a}_{ij}^{(r)}= LeakyRelu\left({q}_i^{(r)}+{k}_j^{(r)}\right) \end{eqnarray*}

Then, the attention coefficients for each relation type are then obtained via the across-relation attention mechanism:


(4) \begin{eqnarray*} {\mathrm{\alpha}}_{ij}^{(r)}=\frac{\mathit{\exp}\left({a}_{ij}^{(r)}\right)}{\sum_{r^{\prime}\in \mathcal{R}}{\sum}_{k\in{\mathcal{N}}_{{\mathscr{r}}^{\prime}}^{\left(\mathscr{i}\right)}}\mathit{\exp}\left({a}_{ik}^{\left({r}^{\prime}\right)}\right)} \end{eqnarray*}

where $\mathcal{R}$ denotes the set of relations, i.e. edge types. ${\mathcal{N}}_{{\mathscr{r}}^{\prime}}^{\left(\mathscr{i}\right)}$ denotes the set of spots connected to node $i$ under relation ${r}^{\prime }$. To avoid overfitting, we employed the dropout strategy on the normalized attention coefficients with dropout rate = 0.3. That is, ${\alpha}_{ij}^{(r)}$ will be set as 0 with a probability of 0.3.

Then, spot $i$ collectively aggregating information from spots connected to it with the neighborhood aggregation step to get the output of layer $l$. We denote the intermediate representation of ${\boldsymbol{x}}_j^{(r)}$ as ${\boldsymbol{h}}_j^{(r)}$: ${\boldsymbol{h}}_j^{(r)}={\boldsymbol{W}}_l^{(r)}{\boldsymbol{x}}_j$. To enhance the discriminative power of the RGAST layer, we further implement the additive cardinality preservation mechanism [23]:


(5) \begin{eqnarray*} {\boldsymbol{x}}_i^{\prime (r)}={\sum_{j\in{\mathcal{N}}_r^{\left(\mathscr{i}\right)}}}{\mathrm{\alpha}}_{ij}^{(r)}{\boldsymbol{h}}_j^{(r)}+\mathcal{W}\odot{\sum_{j\in{\mathcal{N}}_r^{\left(\mathscr{i}\right)}}}{\boldsymbol{h}}_j^{(r)} \end{eqnarray*}

(6) \begin{eqnarray*} {\boldsymbol{x}}_i^{\prime }={\sum_{r\in \mathcal{R}}}{\boldsymbol{x}}_i^{\prime (r)} \end{eqnarray*}

where $\mathcal{W}$ is a nonzero vector $\in{R}^n$, *n* is the output dimension of layer $l$, $\odot$ denotes the elementwise multiplication. The output of encoder is considered as the final spot embedding. In this study, output sizes of two relational graph attention layers are 100 and 32, with latent embedding dimension is set as 32 across all data.

#### Decoder

The decoder reverses the latent embedding back into the original PCA representation of the expression profile. The one-layer linear decoder treats the output of the encoder (denoted by ${\boldsymbol{h}}_i$) as its input and computes the reconstructed result. Specifically, the decoder computes the reconstructed result as follows:


(7) \begin{eqnarray*} \hat{{\boldsymbol{x}}_i}={\boldsymbol{h}}_i{\boldsymbol{W}}^T+\boldsymbol{b} \end{eqnarray*}

where $\boldsymbol{W}$ and $\boldsymbol{b}$ are learnable weight matrix and bias vector.

#### Loss function

The objective of RGAST is to minimize the reconstruction loss of the original PCA profiles as follows:


(8) \begin{eqnarray*} {\sum_{i=1}^N}{\left||\hat{{\boldsymbol{x}}_i}-{\boldsymbol{x}}_i|\right|}_2 \end{eqnarray*}

In all experiments we used Adam optimizer with learning rate = 0.001 and weight decay = 0.0001.

### Deep embedding clustering (optional)

To enhance the compactness of the RGAST embedding, we use an unsupervised deep embedded clustering (DEC) method to iteratively group cells into different clusters [24]. To initialize the cluster centers, we first apply a clustering method to the learned latent representations (such as Louvain, Leiden, Kmeans, etc.). With this initialization, DEC improves the clustering using an unsupervised iterative method of two steps. In the first step, DEC calculates a soft assignment ${q}_{ij}$ of the latent point ${h}_i$ to the cluster center mui using the Student’s t-distribution:


(9) \begin{eqnarray*} {q}_{ij}=\frac{{\left(1+{\left|\left|{h}_i-{\mathrm{\mu}}_j\right|\right|}^2\right)}^{-1}}{\sum_{j^{\prime }}{\left(1+{\left|\left|{h}_i-{\mathrm{\mu}}_{j^{\prime }}\right|\right|}^2\right)}^{-1}} \end{eqnarray*}

In the second step, we iteratively refine the clusters by learning from their high confidence assignments with the help of an auxiliary target distribution $p$ based on ${q}_{ij}$:


(10) \begin{eqnarray*} {p}_{ij}=\frac{q_{ij}^2/{\sum}_i{q}_{ij}}{\sum_{j^{\prime }}\left({q}_{i{j}^{\prime}}^2/{\sum}_i{q}_{i{j}^{\prime }}\right)} \end{eqnarray*}

Based on the soft assignment ${q}_{ij}$ and auxiliary target distribution ${p}_{ij}$, an objective function is defined using the KL divergence:


(11) \begin{eqnarray*} KL\left(P\mid |Q\right)={\sum_i}{\sum_j}{p}_{ij}\,\mathit{\log}\frac{p_{ij}}{q_{ij}} \end{eqnarray*}

Then, the overall loss function is the sum of (8) and (11). The RGAST parameters and cluster centers are then simultaneously optimized using Adam optimizer.

### Scalable training of RGAST

Training RGAST becomes challenging when the number of spots is large, as mini-batch training is not well defined for graph neural networks. Here, we adopted the recently developed DIC strategy for large-scale spatial transcriptomics, which trains on subgraphs and infers on the whole slice [15]. In our Stereo-seq mouse olfactory bulb dataset, we divided the graph into 2 × 2 subgraphs. The dividing point along the *x*-axis is determined by the median of spot locations, which is the same for the *y*-axis.

### Spatial domain detection/clustering analysis

#### Leiden algorithm

We used the Scanpy package to compute the Leiden clustering from the latent embeddings generated from different representation learning methods. Briefly, we used latent embeddings to compute neighborhood graphs with number of neighbors as the recommended default parameter and Euclidean distance as the distance measure. For datasets with ground truth annotation, we set the number of clusters as ground truth, then grid-searching on the Leiden clustering resolutions between 0.0 and 2.5 was performed at intervals of 0.02. For datasets without annotation, resolutions are set as 0.3.

#### Evaluation metrics

##### Adjusted Rand index

The Rand index computes a similarity measure between two clusterings by considering all pairs of samples and counting pairs that are assigned in the same or different clusters in the predicted and true clusterings. The raw RI score is then “adjusted for chance” into the ARI score using the following scheme:


(12) \begin{eqnarray*} ARI=\frac{RI-E(RI)}{\mathit{\max}(RI)-E(RI)} \end{eqnarray*}

To calculate this value, first calculate the contingency table like this:

**Table:** 

	${Y}_1$	${Y}_2$	$\dots$	${Y}_s$	$\mathrm{Sums}$
${X}_1$	${n}_{11}$	${n}_{12}$	$\cdots$	${n}_{1s}$	${a}_1$
${X}_2$	${n}_{21}$	${n}_{22}$	$\cdots$	${n}_{2s}$	${a}_2$
$\vdots$	$\vdots$	$\vdots$	$\ddots$	$\vdots$	$\vdots$
${X}_r$	${n}_{r1}$	${n}_{r1}$	$\cdots$	${n}_{rs}$	${a}_r$
$\mathrm{Sums}$	${b}_1$	${b}_2$	$\cdots$	${b}_s$	

each value in the table represents the number of data points located in both cluster (*Y*) and true class (*X*), and then calculates the ARI value through this table:


(13) \begin{eqnarray*} \overset{\mathrm{Adjust}\ \mathrm{Index}}{\overbrace{ARI}}=\frac{\overset{\mathrm{Index}}{\overbrace{\sum_{ij}\left(\genfrac{}{}{0pt}{}{n_{ij}}{2}\right)}}-\overset{\mathrm{Expected}\ \mathrm{Index}}{\overbrace{\left[{\sum}_i\left(\genfrac{}{}{0pt}{}{a_i}{2}\right){\sum}_j\left(\genfrac{}{}{0pt}{}{b_j}{2}\right)\right]/\left(\genfrac{}{}{0pt}{}{n}{2}\right)}}}{\underset{\operatorname{Max}\ \mathrm{Index}}{\underbrace{\frac{1}{2}\left[{\sum}_i\left(\genfrac{}{}{0pt}{}{a_i}{2}\right)+{\sum}_j\left(\genfrac{}{}{0pt}{}{b_j}{2}\right)\right]}}-\underset{\mathrm{Expected}\ \mathrm{Index}}{\underbrace{\left[{\sum}_i\left(\genfrac{}{}{0pt}{}{a_i}{2}\right){\sum}_j\left(\genfrac{}{}{0pt}{}{b_j}{2}\right)\right]/\left(\genfrac{}{}{0pt}{}{n}{2}\right)}}} \end{eqnarray*}

The ARI is thus ensured to have a value close to 0.0 for random labeling independently of the number of clusters and samples and exactly 1.0 when the clusterings are identical (up to a permutation). The ARI is bounded below by −0.5 for especially discordant clusterings.

##### Silhouettes coefficient

SC is a metric used to evaluate the clustering effectiveness on dataset without ground truth label [25]. ${a}_i$ is denoted as tightness, which represents the average distance between sample $i$ and other samples within same cluster. The average distance between one sample and other samples from different cluster is defined as separation. ${b}_i=\mathit{\min}\left[{b}_{i1},{b}_{i2},\dots, {b}_{ik}\right]$, which is the separation of sample $i$. Then, the SC of sample $i$ is defined as:


(14) \begin{eqnarray*} {s}_i=\frac{b_i-{a}_i}{\mathit{\max}\left({a}_i,{b}_i\right)} \end{eqnarray*}

The SC score is the average of ${s}_i$ ranging from −1 to 1 and a higher score means higher performance.

### Cell–cell communications analysis

Following model training, each relational graph attention convolution (RGATConv) layer generates edge-specific attention scores within the original heterogeneous graph. For our analysis, we specifically utilized the attention scores from the final RGATConv layer to reconstruct the CCC network. To construct a biologically meaningful CCC network, we implemented an attention score thresholding approach. For each individual cell/spot, we: (i) sorted all connecting edges in descending order based on their attention scores; (ii) computed the cumulative sum of attention scores; (iii) retained edges only until the cumulative sum exceeded a predetermined threshold. This selective filtering process effectively preserves the most relevant interactions while eliminating weaker, potentially noisy connections. The resulting pruned graph represents a high-confidence, single-cell resolution CCC network that captures the most significant intercellular communication events.

To investigate the molecular basis of the identified cell–cell interactions, we implemented a quantitative framework to score LR co-expression using the CellChatDB database. For each interacting cell pair predicted by RGAST, the interaction strength of an individual LR pair was calculated by taking the product of the log-transformed expression values of the ligand and the receptor. In cases where ligands or receptors consisted of multiple subunits, we used the mean of all corresponding subunit-pair interaction scores to represent the overall strength of the complex. Furthermore, to assess interactions at a higher biological level, we consolidated the LR-level scores into pathway-level scores. The total interaction strength of a specific pathway between a given cell pair was defined as the mean of the interaction scores of all constituent LR pairs within that pathway. The global activity of each pathway was then determined by averaging these pathway scores across all interacting cell pairs identified by RGAST.

To characterize niche-level signaling direction patterns, we derived spatial vector fields from our reconstructed single-cell CCC network. Given the communication strength matrix $\boldsymbol{S}\in{\mathbb{R}}_{+}^{n_s\times{n}_s}$ (we use reconstructed attention weights for overall analysis and co-expression score for specific LR pair or pathway analysis), where ${\boldsymbol{S}}_{ij}$ represents the directed signaling strength from spot $i$ to spot $j$, we computed two distinct vector fields: signal-sending vector field (${\boldsymbol{V}}^s$) and signal-receiving vector field (${\boldsymbol{V}}^r$). For each spot $i$, we define:


\begin{eqnarray*} && {\boldsymbol{V}}_i^s=\left({\sum}_j{\boldsymbol{S}}_{i,j}\right)\times \mathcal{N}\left({\sum}_{j\in{N}_i^s}{\boldsymbol{S}}_{i,j}\left({\boldsymbol{x}}_j-{\boldsymbol{x}}_i\right)\right)\\&& {\boldsymbol{V}}_i^r=\left({\sum}_j{\boldsymbol{S}}_{j,i}\right)\times \mathcal{N}\left({\sum}_{j\in{N}_i^r}{\boldsymbol{S}}_{j,i}\left({\boldsymbol{x}}_i-{\boldsymbol{x}}_j\right)\right),\end{eqnarray*}

where $\mathcal{N}\left(\boldsymbol{x}\right)=\boldsymbol{x}\Vert \boldsymbol{x}$ denotes the normalization operator, ${N}_i^s$ represents the index set of top k signal-sending neighbors for spot $i$ (highest values in row $i$ of $\boldsymbol{S}$), ${N}_i^r$ represents the index set of top k signal-receiving neighbors for spot $i$ (highest values in column $i$ of $\boldsymbol{S}$). ${\boldsymbol{x}}_i\in{\mathbb{R}}^2$ denotes the spatial coordinates of spot $i$.

To derive cell type-level CCC metrics, we implemented a normalized counting approach that accounts for cellular composition biases. For each pairwise interaction between cell types A and B, we: (i) enumerated all intercellular connections (edges) between the two cell types; (ii) normalized the raw connection strength by the product of their respective cell population sizes (${N}_A\times{N}_B$); (iii) defined this normalized value as the communication score ($C{S}_{AB}$): $C{S}_{AB}$ = (Connection strength between A and B) / (${N}_A\times{N}_B$). The communication score mitigates the confounding effects of unequal cell type abundances and provides an unbiased metric for comparing communication strengths across different cell type pairs.

### Benchmarking different CCC methods

In real data, single-cell level CCC network ground truth does not exist. So the benchmark is based on curated LR-driven simulated dataset. To make the simulated data more similar to real-world conditions, our simulation strategy involves perturbing the real-world data. First, we defined a set of curated cell type-specific LR interaction rules which has been validated by previous research (e.g. Excitatory → Inhibitory via Oxt-Oxtr. Full list can be found at Supplementary Table S2). For each LR pair, we first identified cells of the corresponding sender and receiver cell types from the spatial transcriptomics dataset. We then randomly selected a subset of these sender and receiver cells as candidate interactors. To simulate realistic spatial communication, we modeled the interaction probability ${P}_{ij}$ between a sender cell $i$ and a receiver cell $j$ using a squared decay function: ${P}_{ij}\propto 1/{d}_{ij}^2$. Each sender cell was then paired with several receiver cells sampled from the candidate pool based on these distance-weighted probabilities. Subsequently, we simulated the functional impact of CCC by amplifying the expression levels of the ligand (in sender cells) and receptor (in receiver cells) using a normally distributed amplification factor. We recorded the ground-truth interaction matrix (with amplification intensity) and binary interaction matrix (presence/absence of CCC) to quantify true positive interactions, and added Gaussian noise to the expression matrix to mimic biological variability in real datasets. Using the ground truth CCC matrix as the ground truth, we computed standard binary classification metrics (e.g. precision, recall, F1-score, and accuracy) to evaluate model’s performance.

### Spatially variable genes detection

#### Detection of SVGs

In this study, we employ the same detection method as that used in SpaGCN to identify SVGs enriched in each spatial domain generated by different methods. We recognize that some genes may be expressed in multiple but disconnected domains. Therefore, instead of conducting differential expression (DE) analysis using spots from a target domain versus all other spots, we first select spots to form a neighboring set around the target domain. Our aim is to detect genes that are highly expressed in the target domain but not expressed or expressed at low levels in neighboring spots. To achieve this, we draw a circle with a predetermined radius around each spot in the target domain, and spots from non-target domains within the circle are considered its neighbors. The radius is set such that each spot in the target domain has an average of approximately 10 neighbors. Next, we collect all the neighbors of spots in the target domain to form a neighboring set. For each non-target domain, if more than 50% of its spots are in the neighboring set, we select it as a neighboring domain. This criterion is in place to avoid cases where a domain is selected as a neighboring domain, but only a small proportion of its spots are adjacent to the target domain. Once neighboring domains are determined, we perform DE analysis between spots in the target domain and the neighboring domain(s) using the Wilcoxon rank-sum test. Genes with a false discovery rate (FDR)-adjusted *P*-value <.05 are selected as SVGs. To ensure only genes with enriched expression patterns in the target domain are selected, we require a gene to meet the following three criteria: (i) the percentage of spots expressing the gene in the target domain, that is, in-fraction, is >80%; (ii) for each neighboring domain, the ratio of the percentages of spots expressing the gene in the target domain and the neighboring domain(s), that is, in/out fraction ratio, is >1; and (iii) the expression fold change between the target and neighboring domain(s) is >1.5.

#### Evaluation metrices

Gene expressions at different locations may not be independent. For example, the expression levels of a gene at nearby locations may be closer in value than expression levels at locations that are farther apart. This phenomenon is called spatial autocorrelation, which measures the correlation of a variable with itself through space. To evaluate whether the detected SVGs exhibit an organized spatial expression pattern, we used Moran’s I and Geary’s C, two commonly used statistics to quantify the degree of spatial autocorrelation of gene expression.

#### Moran’s I statistic

Moran’s I metric is a correlation coefficient that measures the overall spatial autocorrelation of a dataset [26]. Essentially, it quantifies how similar one spot is to other spots surrounding it for a given gene. If the spots are attracted or repelled by each other, it suggests that they are not independent, and the presence of autocorrelation indicates a spatial pattern of gene expression. The Moran’s I value ranges from −1 to 1, where a value close to 1 indicates a clear spatial pattern, a value close to 0 indicates random spatial expression, and a value close to −1 indicates a chessboard-like pattern. To evaluate the spatial variability of a given gene, we calculate the Moran’s I using the following formula:


(15) \begin{eqnarray*} I=\frac{N}{W}\frac{\sum_i{\sum}_j\left[{w}_{ij}\left({x}_i-\overline{x}\right)\left({x}_j-\overline{x}\right)\right]}{\sum_i{\left({x}_i-\overline{x}\right)}^2} \end{eqnarray*}

where ${x}_i$ and ${x}_j$ are the gene expression of spots $i$ and $j$, $\overline{x}$ is the mean expression of the gene, $N$ is the total number of spots, ${w}_{ij}$ is the spatial weight between spots $i$ and $j$ calculated using the 2D spatial coordinates of the spots, and $W$ is the sum of ${w}_{ij}$. To select the k nearest neighbors for each spot, we use spatial coordinates. The Moran’s I statistic is robust to the choice of k, and we set it to 5 in our analysis. We assign ${w}_{ij}$ = 1 if spot $j$ is in the nearest neighbors of spot $i$, and ${w}_{ij}$ = 0 otherwise.

#### Geary’s C statistic

Geary’s C statistic is another measure of spatial autocorrelation, which is a statistical method used to examine the degree to which neighboring observations in a dataset are similar to each other for a given gene [27]. In other words, it measures the extent to which spots that are close to each other in space are more similar or dissimilar than those that are farther apart with respect to the gene expression. Geary’s C is calculated using the following formula:


(16) \begin{eqnarray*} C=\frac{N}{2W}\frac{\sum_i{\sum}_j\left[{w}_{ij}{\left({x}_i-{x}_j\right)}^2\right]}{\sum_i{\left({x}_i-\overline{x}\right)}^2} \end{eqnarray*}

The value of Geary’s C ranges from 0 to 2, with a value of 1 indicating no spatial autocorrelation, values less than 1 indicating positive spatial autocorrelation (i.e. neighboring expressions are more similar than expected by chance), and values greater than 1 indicating negative spatial autocorrelation (i.e. neighboring expressions are more dissimilar than expected by chance).

### Trajectory and pseudotime inference

#### PAGA graph and UMAPs

We also used the Scanpy package to compute the partition-based graph abstraction (PAGA) and UMAP from embeddings generated from different methods. After using latent embeddings to compute neighborhood graphs, UMAP algorithm was conducted based on the neighborhood graphs. Finally, PAGA was applied to quantify the connectivity between different layers based on the UMAP representations.

#### Monocle3

For SEDR, STAGATE, conST, and RGAST, we first extracted the low-dimensional embedding and then used the embedding to replace the default PCs used in the Monocle3 package. We then ran Monocle3 on UMAPs generated by the latent embedding using the recommended parameters and randomly set a spot in WM as the starting point to generate the pseudo-time.

#### Cross-sectional 3D RGAST model

All current ST technologies capture gene expression patterns within the context of 2D tissue sections, which limits the accurate representation of 3D spatial information in real-world samples. A conventional approach to address this limitation is by reconstructing gene expressions in 3D space through the stacking of consecutive 2D sections [28, 29]. However, the presence of batch effects between sections poses a challenge in extracting meaningful 3D spatial patterns. Here, we proposed a 3D RGAST model that incorporates both the 2D relationships within each section and the spatial neighborhood relationships between adjacent sections to mitigate the impact of batch effects. If the presence of minimal batch effects between different sections is confirmed, gene expression similarity can also be applied across sections. Specifically, the spatial neighborhoods between adjacent sections are constructed based on aligned coordinates and a predefined radius. The underlying principle behind the use of 3D spatial neighborhoods lies in the assumption that biological differences between consecutive sections should demonstrate continuity. By enhancing the similarity between adjacent sections, we aim to eliminate discontinuous independent technical noises.

We introduced LTA as a metric to quantify the cross-slice continuity of the inferred 3D domains. Specifically, LTA measures how well the cluster identity of a cell in section ${z}_{i+1}$ can be predicted by its spatial neighbors in the adjacent section ${z}_i$. For each cell, we identified its $k$ nearest spatial neighbors in the preceding slice and predicted its label using majority voting. High accuracy implies that the 3D reconstruction successfully aligns spatially corresponding structures across sections without abrupt disruptions.

## Results

### Overview of the RGAST model

**Figure 1:**
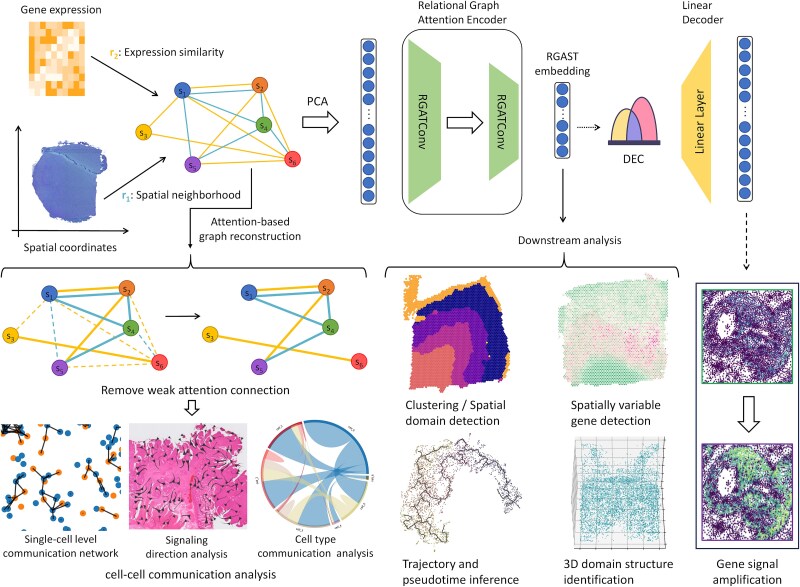
Overview of the RGAST model. RGAST constructs a heterogeneous graph using both spatial information and gene expressions. The expression after dimensionality reduction by PCA of each spot is first transformed into a d-dimensional latent embedding by a relational graph attention encoder and then reversed back into a reconstructed expression profile via a linear decoder. An optional unsupervised deep clustering method is then employed to enhance the compactness of the learned latent representation. After training, the graph can be reconstructed by removing edges with low attention score. The reconstructed graph is then used to conduct multi-scale CCC analysis. The latent embeddings can be applied to many downstream analyses like identifying spatial domains, SVG detection, trajectory inference, and extracting 3D spatial domains. Simultaneously, the decoder-generated expression profile is leveraged to amplify critical biological signals.

The spatial distribution of transcriptional expression in tissues is critical for elucidating biological functions and characterizing interactive biological networks [30]. ST data provides valuable location-specific information that can be leveraged effectively. Notably, certain tissue spots, despite being physically distant, may exhibit long-range communication and similar expression patterns due to their participation in spatially regulated processes such as diffusion or signaling [31]. In the RGAST model, we construct a heterogeneous graph by considering two distinct relationships (Materials and Methods). The first is based on gene expression similarity between spots, while the second is determined by spatial proximity, accounting for their relative physical locations (Fig. 1). By integrating both relationships, we employ a relation-aware attention mechanism that adaptively learns edge weights between spots, dynamically determining the importance of neighboring spots relative to a target spot. These learned weights reflect the communication potential between two cells/spots and are used to update the target spot’s representation through weighted aggregation of information from connected spots.

Using a relational graph attention auto-encoder, RGAST learns CCC pattern and low-dimensional latent embeddings of each spot that encode both spatial and gene expression information (Fig. 1). First, the expression profiles of each spot undergo dimensionality reduction via principal component analysis (PCA). An encoder then maps the reduced expression into a d-dimensional latent embedding, which is reconstructed into an expression profile using a linear decoder (Materials and Methods). Optionally, an unsupervised deep clustering method can be applied to improve the compactness of the learned latent representation [24] (Materials and Methods). Upon convergence, the learned attention scores enable the reconstruction of a CCC graph by filtering out low-confidence edges. The resulting CCC atlas can be aggregated to perform comprehensive CCC analysis (Materials and Methods). Furthermore, the derived latent embeddings can enhance various downstream analyses, including spatial domain identification, SVG detection, trajectory inference, and 3D spatial domain extraction. Concurrently, the reconstructed expression profile can also be utilized to amplify biologically significant gene signals, a capability established by the HERGAST model [15].

### RGAST improves spatial domain detection in various ST technologies with scalability

**Figure 2:**
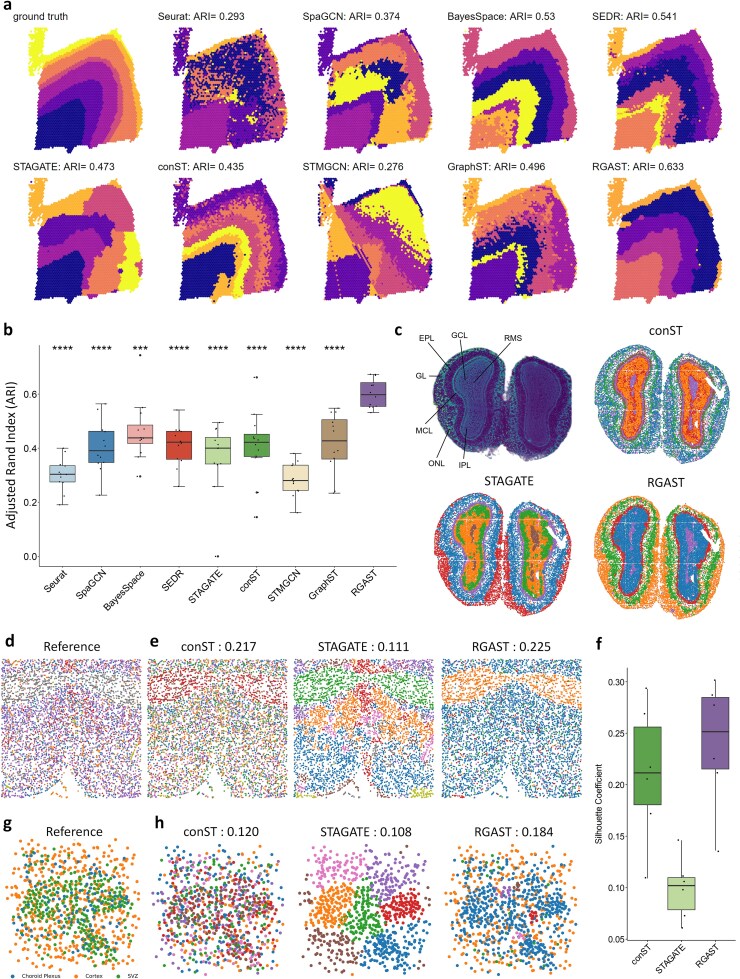
RGAST improves spatial domain detection in various ST technologies. (a) Clustering results of different methods in the 10× Visium DLPFC section 151675 dataset. (b) Boxplot of clustering accuracy for 9 methods across all 12 sections of the 10× Visium DLPFC datasets, measured in terms of ARI scores. The reference method (“RGAST”) is compared against other methods using a paired Wilcoxon signed-rank test (one-sided, alternative hypothesis: ARI < RGAST). Benjamini–Hochberg adjusted significance levels are denoted as: (^*^^*^^*^^*^, *P* < .0001; ^*^^*^^*^, *P* < .001; ^*^^*^, *P* < .01; ^*^, *P* < .05; “ns”, not significant). In the boxplot, the center line, box limits, and whiskers represent the median, upper and lower quartiles, and 1.5$\times$ times interquartile range, respectively. These parameters are consistent across all boxplots in this paper. (c) Spatial domains generated by Leiden clustering with a maximum of seven clusters on the low-dimensional conST, STAGATE, and RGAST embeddings in the Stereo-seq mouse olfactory bulb tissue section. Top left panel is the laminar organization of the mouse olfactory bulb annotated in the DAPI-stained image generated by Stereo-seq. RMS, rostral migratory stream; GCL, granule cell layer; IPL, internal plexiform layer; MCL, mitral cell layer; EPL, external plexiform layer; ONL, olfactory nerve layer. (d) Cell annotation by Moffitt et al. based on scRNA-seq profiling. (e) Spatial domains generated by different methods in the MERFISH data of section Bregma +0.26. The number in the title represents the SC score of the different methods, which is consistent with (h). (f) Boxplot of clustering accuracy for different methods across all six sections of the MERFISH datasets, measured in terms of SC scores. (g) Cell annotation by area, including the choroid plexus, SVZ, and cortex area in the mouse cortex SeqFISH+ dataset. (h) Spatial domains generated by different methods in the mouse cortex SeqFISH+ dataset.

To evaluate RGAST’s spatial clustering performance, we first applied it to ST datasets generated using multiple technologies. Our initial benchmark utilized the 10× Visium platform, analyzing spatial expression profiles from 12 human dorsolateral prefrontal cortex (DLPFC) tissue sections [18]. Maynard et al. manually annotated the DLPFC layers and white matter (WM) based on morphological features and gene markers, which we adopted as the ground truth. To compare the clustering accuracy of RGAST with other methods, we employed the adjusted Rand index (ARI) (Materials and Methods) and compared it with the non-spatial clustering method Seurat and seven recently developed spatial clustering approaches: SpaGCN [7], BayesSpace [8], SEDR [5], STAGATE [9], conST [6], STMGCN [32], and GraphST [10]. To ensure a fair comparison, we set the maximum number of clusters for each method the same as the ground truth label and used the recommended default parameters for all other methods. For methods that generated latent embedding (i.e. SEDR, STAGATE, cost, GraphST, and RGAST), we utilized the Leiden clustering algorithm (Materials and Methods). The results demonstrated that RGAST effectively identified the expected cortical layer structures and achieved significantly better performance across all slices (Fig. 2b and Supplementary Figs S1–S11). For instance, in DLPFC section 151675, RGAST clearly delineated the layer borders and achieved the best clustering accuracy (ARI = 0.633, Fig. 2a). In contrast, the clustering assignment of the non-spatial method Seurat was discontinuous, with many outliers that impeded its clustering accuracy. Not surprisingly, algorithms that leveraged spatial information substantially outperformed the non-spatial clustering method Seurat (Fig. 2b). These findings underscore the superiority of RGAST in spatial domain identification and the necessity to incorporate spatial information in spatial clustering analysis.

Given the central role of graph topology in our model, we further conducted comprehensive analyses to evaluate the contribution of different edge types, parameter sensitivity, and model robustness to structural noise using the DLPFC datasets. First, to elucidate the necessity of the heterogeneous graph architecture, we performed an ablation study by decoupling the spatial and expression edges. We compared the full RGAST model against two variants: one using only gene expression similarity edges and another using only spatial proximity edges. Across all 12 DLPFC slices, the full heterogeneous graph consistently outperformed both single-relation variants (Supplementary Fig. S12). Specifically, models relying solely on expression edges often produced fragmented clusters lacking spatial continuity, while those using only spatial edges tended to over-smooth boundaries, missing fine-grained biological heterogeneity. This confirms that RGAST does not merely smooth gene expression but effectively integrates complementary information from both modalities to capture complex tissue patterns. Second, we examined the sensitivity of RGAST to the hyperparameters governing graph construction: the number of neighbors for the spatial graph (${k}_s$) and the expression graph (${k}_e$). We performed a grid search on DLPFC section 151675, varying both ${k}_s$ and ${k}_e$ from 1 to 12. The results revealed that optimal performance is achieved when both neighbor counts are balanced and moderate (Supplementary Fig. S13). Extremely sparse connectivity in either graph degraded performance, further supporting the synergy of integrating both relationship types. Finally, to assess the model’s robustness to data-specific noise and structural perturbations, we conducted a stringent “hold-out” evaluation by randomly dropping 0% to 50% of the data points from the raw data of all 12 DLPFC slices. RGAST demonstrated remarkable stability, maintaining high ARI with minimal degradation even when up to 40% of the data was removed (Supplementary Fig. S14). A recognizable drop in performance was observed only under extreme data loss (data loss rates >40%), indicating that the model remains stable under substantial node removal and is not overly sensitive to moderate perturbations of the observed graph structure.

Next, we compared the performance of RGAST with conST and STAGATE, which have been demonstrated to be superior to other techniques [6, 9], using the Stereo-seq Mouse olfactory bulb dataset. This emerging spatial omics technology can achieve subcellular spatial resolution using DNA nanoball patterned array chips, and the data used here were binned into a cellular-level resolution of ~14 μm [3]. Fu et al. annotated the laminar organization of the coronal mouse olfactory bulb in the DAPI-stained image [5] (Fig. 2c). Since this dataset contains ~20 000 spots, we employed a recently developed Divide-Iterate-Conquer (DIC) strategy to prevent Graphics Processing Unit (GPU) memory exhaustion during training [15], thereby enhancing the scalability of RGAST (Materials and Methods). Compared to the clusters identified by conST, those identified using both STAGATE and RGAST embeddings more accurately reflected the laminar organization and corresponded well to the annotated layers. However, STAGATE erroneously separated the GCL into two clusters, whereas RGAST did not (Fig. 2c). Furthermore, the five main layer structures identified by RGAST were all validated by the corresponding marker genes (Supplementary Fig. S15).

We also conducted experiments using Mouse hypothalamic preoptic MERFISH and Mouse cortex seqFISH+ data, which were captured at a higher resolution to achieve single-cell resolution, but no morphology data were available. Since no gold standard was available, we used Silhouette Coefficient (SC) to evaluate the clustering performance of each method. For the MERFISH datasets, Moffitt et al. annotated the cell types based on scRNA-seq expression data [16] (Fig. 2d). The results showed that RGAST consistently outperformed the other methods in all six slices, with STAGATE far behind (Fig. 2e and f and Supplementary Figs S16–S20). For example, in slice Bregma +0.26, RGAST was more consistent with the clustering results generated by the expression profile (Fig. 2e). In the Mouse cortex seqFISH+ dataset, the spots were annotated as the choroid plexus, subventricular zone (SVZ), and cortex area [20] (Fig. 2g). RGAST still demonstrated better performance than the other two methods (Fig. 2h). It is noteworthy that STAGATE appeared to overuse the spatial neighborhood relationship, as it showed obvious clustering characteristics of block aggregation in such data with discrete cell type distribution (Fig. 2e and h).

### Validation and application of RGAST in deciphering spatially resolved cell–cell communication networks

**Figure 3:**
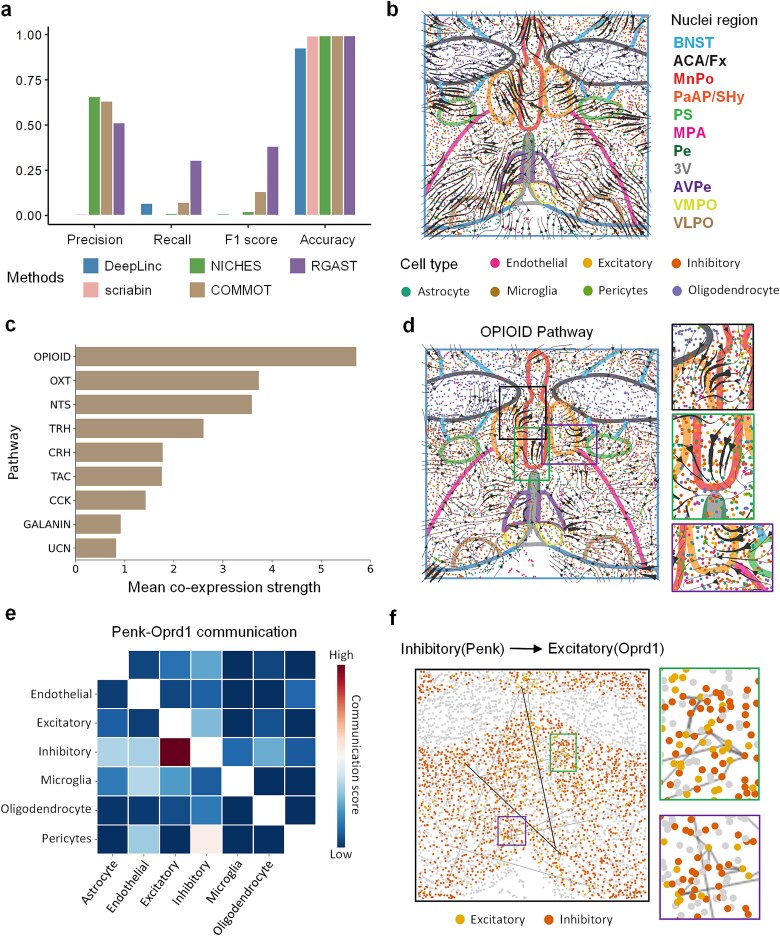
Multi-scale CCC analysis in the mouse hypothalamic preoptic region. (a) Performance of different methods in constructing single-cell communications network based on simulated data. (b) Niche-level communication stream plot of the Bregma +0.26 slice. The cell type annotation and hypothalamic nuclei segmentation are adopted from Moffitt et al. and are colored identically to the nuclei abbreviations listed to the right. BNST, bed nucleus of the stria terminalis; ACA, anterior commissure; Fx, fornix; MnPO, median preoptic nucleus; PaAP, paraventricular hypothalamic nucleus, anterior parvicellular; SHy, septohypothalamic nucleus; PS, parastrial nucleus; MPA, medial preoptic area; Pe, periventricular hypothalamic nucleus; 3V, third ventricle periventricular nucleus; AvPe, anteroventral; VMPO, ventromedial preoptic nucleus; VLPO, ventrolateral preoptic nucleus. (c) Mean co-expression scores of the identified pathways based on RGAST’s inferred CCC network. (d) Communication stream plot of OPIOID pathway. Right panels are the zoomed-in view of the selected regions in the left panel. The cell type annotation and hypothalamic nuclei segmentation are identical to (c). (e) Heatmap of the communication score of ligand–receptor pair Penk-Oprd1 between different cell types. (f) Spatial *in-situ* visualization of the Penk-Oprd1 communication between Inhibitory and Excitatory. Two long-range communications are highlighted in the plot. Right panels are the zoomed-in view of the selected regions in the left panel.

To assess RGAST’s capability in deciphering CCC at single-cell resolution, we first applied it to a curated ligand–receptor (LR)-driven simulated dataset (Materials and Methods). For benchmarking, we compared RGAST against several SOTA methods specifically designed for single-cell CCC inference: DeepLinc [11], Scriabin [12], NICHES [13], and COMMOT [14]. Consistent with their methodological nature of heavy reliance on LR co-expression patterns to infer CCC, NICHES and COMMOT exhibited higher precision values (Fig. 3a). However, RGAST achieved substantially higher recall and F1 scores, indicating that it can capture more true positive CCCs while maintaining a balanced trade-off between precision and recall. Collectively, these results confirm that RGAST still outperforms other methods in overall performance for CCC detection in spatial transcriptomics data. Among the benchmarked methods, RGAST, Scriabin, NICHES, and COMMOT maintained near-perfect accuracy, whereas DeepLinc exhibited comparatively lower performance. This discrepancy can be largely attributed to DeepLinc’s over-sensitivity add-on paradigm in graph reconstructions, which led to an inflated detection of spurious cell connections.

To explore the relevance of using attention weights learned by RGAST as a contributing factor for CCC inference, we conducted two complementary analyses based on the same curated LR-driven simulated dataset. First, we quantified the association between the attention weights learned by the trained RGAST model and the ground-truth CCC interaction intensity using the Spearman’s rank correlation coefficient. Our results revealed a moderate, though statistically significant, positive correlation (Spearman’s $\mathrm{\rho}$ = 0.645, Supplementary Fig. S21a). While this indicates that attention weights alone are not fully predictive of the exact simulated ground truth intensity, it suggests that after integrating raw gene expression profiles, expression similarity, and spatial neighborhood relationships, the attention mechanism captures features that partially reflect underlying LR-mediated intercellular communication. Second, we assessed the impact of varying attention weight cutoffs (ranging from 0.1 to 1.0 with 0.1 increments) on the strength of this correlation to evaluate model robustness and derive practical parameter guidelines. Overall, the Spearman’s correlation coefficient remained relatively stable at approximately 0.6 for cutoffs greater than 0.5, with a marked decline observed primarily at extreme low thresholds (Supplementary Fig. S21b). This pattern indicates that RGAST’s attention mechanism maintains a consistent, albeit partial, association with underlying CCC signals, and its performance in prioritizing potential CCC candidates is robust to moderate variations in threshold selection.

We next conducted a detailed multi-scale CCC analysis on the MERFISH Bregma +0.26 slice. At the overall cell-type level, oligodendrocyte-microglia interactions were the most prominent (Supplementary Fig. S22a), aligning with their well-documented roles in myelin maintenance and neuroinflammatory responses [33–35]. In contrast, oligodendrocyte-pericyte interactions were notably sparse (Supplementary Fig. S22a). Astrocytes emerged as central hubs in the CCC network, participating in the majority of interactions (Supplementary Fig. S22a). This pan-cellular connectivity corroborates astrocytes’ function as metabolic and immune integrators [36]. This observation was further supported at single-cell resolution: astrocytes exhibited widespread spatial distribution, enabling frequent short-range interactions with diverse cell types (Supplementary Fig. S22b), whereas oligodendrocytes were confined to specific niches and primarily engaged in homotypic interactions (Supplementary Fig. S22c).

Niche-level analysis further uncovered a directional signaling from peripheral hypothalamic nuclei (BNST, PS, MPA, VLPO) to central regions (MnPO, 3V, VMPO) (Fig. 3b). To further elucidate the underlying molecular mechanism in this result, we conducted ligand–receptor co-expression analysis of each interacted cell pair and got aggregated pathway communication scores (Materials and Methods). These identified pathways are all related to neuropeptides and hormones, which play key neuronal signal regulatory roles in the nervous system (Fig. 3c). This indicates that MnPO and VMPO, as the central nuclei of preoptic area (POA), may be the core hub for integrating multiple physiological and behavioral signals. This organizational framework is supported by recent single-cell transcriptomic evidence showing that POA neurons establish functional specialization during embryonic development, with inter-regional communication networks maturing postnatally through progressive refinement of signaling pathways [37].

Next, we examined the OPIOID signaling pathway, which exhibited the highest communication score in our analysis (Fig. 3c). Previous studies have demonstrated the presence of enkephalinergic nerve fibers projecting from the MPA and MnPo to the PaAP and Pe region [38]. In the pathway-level communication direction, we can see salient signal from MnPo to PaAP (Fig. 3d, black box), from MnPo to Pe (Fig. 3d, green box), and from MPA to PaAP (Fig. 3d, purple box), thereby confirming these established findings. Furthermore, we investigated the Penk-Oprd1 ligand–receptor interaction within the OPIOID pathway to elucidate the communication patterns between different cell groups. Interestingly, the signaling from inhibitory to excitatory neurons was particularly strong, while interactions between other cell types were negligible (Fig. 3e). This result is consistent with well-established conclusions. For instance, Tricoire et al. identified SST inhibitory neurons as the source of Penk expression [39]. Additionally, Svingos et al. provided physical evidence that OPRD1 receptors are localized on the dendrites of excitatory pyramidal neurons [40]. Complementing these findings, Glaum et al. demonstrated that the activation of OPRD1 receptors directly inhibits the functional effects of excitatory glutamate release [41]. Finally, our *in situ* visualization of this specific Inhibitory (Penk)-to-Excitatory (Oprd1) signaling revealed that it encompasses not only short-range local networks (Fig. 3f, right panels) but also long-range communications across the entire tissue slice (Fig. 3f, left panel). These long-range interactions cannot be detected by distance-constrained CCC analysis methods typically applied to spatial transcriptomics data. The identification of these known neuroglial interaction patterns not only validates RGAST’s spatial resolution but also confirms its ability to reconstruct functional hierarchies within complex neural circuits.

To assess its performance in a highly complex system, we applied RGAST to a multiplexed breast cancer dataset (Supplementary Fig. S23). The analysis successfully recapitulated established biological phenomena, such as strong T-cell-B-cell collaboration and weak stromal–epithelial interaction [42, 43] (Supplementary Fig. S23a). Moreover, it revealed distinct communication patterns, identifying epithelial cells as primarily engaging in self-interaction [44] (Supplementary Fig. S23a and c) while T-cells act as central immune coordinators with extensive connections to other cell types [45] (Supplementary Fig. S23a and d). Critically, RGAST uncovered a prominent directional signaling axis from the invasive cancer periphery towards fibrous and fatty tissue regions, a spatial progression that mirrors the multistep cascade of metastasis, involving stromal remodeling and metabolic adaptation [46] (Supplementary Fig. S23b). The method also identified significant long-range interactions between T-cells and distal macrophages, endothelial, and B-cells (Supplementary Fig. S23e). This aligns with studies demonstrating cytokine-mediated distal communication (e.g. CCL5-CCR5 axis) and exosome trafficking across tumor niches [47, 48]. These findings underscore the utility of RGAST in capturing both local and systemic communication networks within spatially complex tumors.

### Benchmarking reveals the superiority of RGAST for SVG identification

SVGs exhibit varying expression patterns across distinct spatial locations. Identifying SVGs can provide valuable insights into the biological processes that underlie the development and function of tissues and organs. To this end, we incorporated SpatialDE [49], an embedding-independent method based on Gaussian process regression, alongside with SpaGCN, conST, and STAGATE into our benchmarking. We compared the performance of these methods to that of RGAST using datasets generated by different ST techniques.

**Figure 4:**
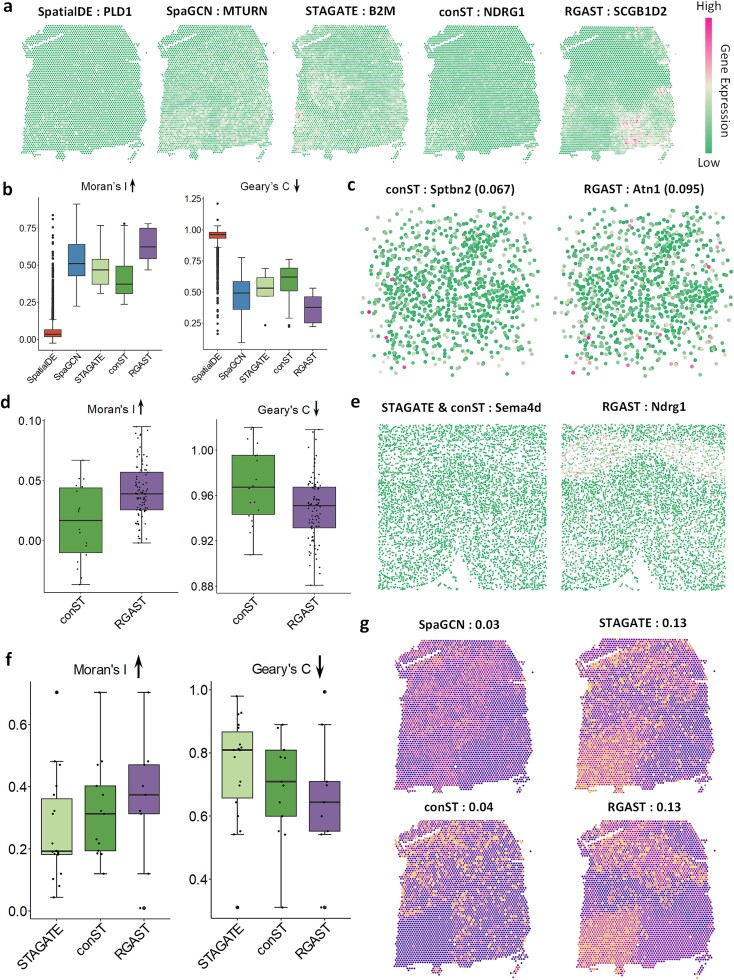
RGAST assists in identifying spatially variable genes. (a) Examples of the spatial distribution of SVGs exclusively detected by SpatialDE, SpaGCN, STAGATE, conST, and RGAST, respectively, in the DLPFC dataset section 151674. (b) Boxplot of Moran’s I and Geary’s C statistics for all SVGs detected by each method in the DLPFC dataset section 151674. Upward arrow means higher value is better, while downward arrow means lower value is better. (c) Spatial distribution of SVGs detected by conST and RGAST with the highest Moran’s I in mouse cortex SeqFISH+ data. STAGATE failed to detect any SVGs. (d) Boxplot of Moran’s I and Geary’s C statistics for all SVGs detected by each method in mouse cortex SeqFISH+ data. (e) Spatial distribution of SVGs detected by STAGATE conST and RGAST, respectively, in MERFISH data slice Bregma +0.26 with a similar target domain. (f) Boxplot of Moran’s I and Geary’s C statistics for all SVGs detected by each method in MERFISH data slice Bregma +0.26. (g) Clustering results using only four SVGs detected by each method as features. The number in the title denotes the ARI score.

We first used section 151674 of the DLPFC dataset from the 10× Genomics Visium platform as our benchmark dataset. To evaluate the quality of the identified SVGs, we employed Moran’s I and Geary’s C statistics to quantify the spatial autocorrelation. In the context of tissue architecture, high spatial autocorrelation serves as a robust proxy for spatial coherence and structural continuity. Our results demonstrated that SVGs exclusively detected by RGAST exhibited clear spatial distribution patterns consistent with anatomical layers. In contrast, genes identified by the embedding-independent method SpatialDE often showed scattered or ambiguous patterns, resulting in the lowest Moran’s I and highest Geary’s C scores (Fig. 4a and b). Benchmarking across all 12 slices, the SVGs identified by RGAST consistently achieved the highest (or second highest) Moran’s I and the lowest (or second lowest) Geary’s C, with SpatialDE consistently being the worst performance (Supplementary Fig. S24). This suggests that pure variance-based detection may capture stochastic noise, whereas RGAST prioritizes genes with spatially coherent structures. Overall, SVGs identified by RGAST showed significantly higher spatial coherence than those identified by other methods (Fig. 4a and b).

Due to the absence of pathological images in the MERFISH and SeqFISH+ datasets, SpaGCN was not included in our follow-up comparison. SpatialDE was also removed for the consistently lowest performance. Interestingly, STAGATE failed to detect any SVGs in the mouse cortex SeqFISH+ dataset, also indicating an unclear spatial domain pattern in this dataset (Fig. 4c). To address this issue, we visualized the SVGs detected by RGAST and conST with the highest Moran’s I, which revealed the superior performance of RGAST (Fig. 4c). In fact, RGAST outperformed conST even when we considered all the SVGs detected by each method (Fig. 4d). For the MERFISH data, we used slice Bregma +0.26 as our benchmarking dataset and found that SVGs identified by RGAST displayed a clearer spatial distribution pattern, even when the target domain was similar (Fig. 4e). This was confirmed by our quantitative comparison as well (Fig. 4f). Lastly, to evaluate the representational ability of SVGs detected by each method, we used the SVGs as features to conduct clustering on the DLPFC data, with ARI as our evaluation criterion. For a fair comparison, we randomly selected four SVGs for each method, since STAGATE only detects four SVGs. Our results showed that RGAST and STAGATE performed similarly and significantly better than SpaGCN and conST (Fig. 4g).

### Revealing developmental trajectories more accurately with RGAST

Trajectory inference is a method used to infer the pattern of cells in dynamic developmental processes, with pseudotime representing the progression through this process. Improving the quality of the embedding method can significantly enhance the accuracy and resolution of trajectory inference results [50]. In this study, we selected the DLPFC dataset for trajectory and pseudotime inference. We used section 151676 as the benchmarking data and compared three methods—SEDR, STAGATE, and conST—with RGAST, as these methods have shown superior performance in trajectory inference analysis [5, 6, 9]. To validate the quality of the learned embeddings, we used Monocle3 [51] to produce trajectory and pseudotime inference results with embeddings generated from different methods. For pseudotime inference, we selected WM as the starting point (Materials and Methods).

**Figure 5:**
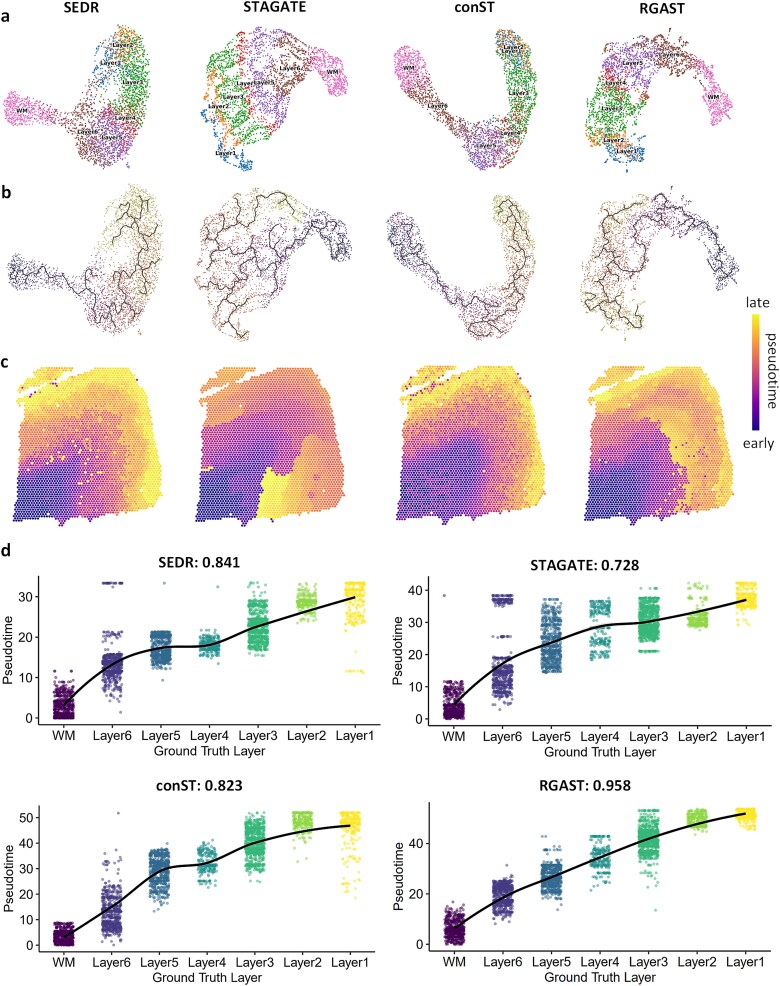
RGAST reveals cell trajectories more accurately. (a) UMAP visualizations colored by different layers generated by SEDR, STAGATE, conST, and RGAST embeddings, respectively, in the DLPFC section 151676. The order of plots for different methods in (b) and (c) is consistent with (a). (b) UMAP visualizations colored by pseudotime inferred from SEDR, STAGATE, conST, and RGAST embeddings, respectively. (c) Pseudotime inference results from different methods depicted on the spatial physical context. (d) Quantitative evaluation of trajectory inference across cortical layers in the DLPFC dataset. Scatter plots showing the relationship between the ground-truth anatomical layers (*x*-axis) and the inferred pseudotime (*y*-axis) based on embeddings from different methods. The black curves represent the local regression (LOESS) fit. Numbers at the title of each panel represent the Spearman rank correlation coefficients.

Based on the results, the uniform manifold approximation and projection (UMAP) visualization generated by the SEDR embedding appeared as a circular structure in which layers 1 and 6 are connected to each other (Fig. 5a). In contrast, the conST and RGAST embeddings showed a streamlined expansion of different layers, while the UMAP visualization of the STAGATE embedding appears clumped together without an obvious linear structure (Fig. 5a). In the inferred development trajectory and pseudotime, STAGATE exhibited too many branching points, and the visualization in the spatial context confirmed the incorrect developmental trajectory inferred by STAGATE (Fig. 5b and c). The inferred pseudotime in this context serves as a proxy for cortical depth or the continuous transcriptomic gradient across anatomical layers. While the pseudotime inferred by the other three methods reflects the correct “inside-out” depth ordering of cortical layers, the pseudotime inferred by SEDR and conST is not as smooth as RGAST and exhibits more outliers in each layer structure (Fig. 5b and c). Therefore, our results suggest that RGAST can reveal cell trajectories more accurately than the other methods.

To quantify the performance of each method in trajectory analysis, we mapped the discrete layer annotations to ordinal numeric values and calculated the Spearman rank correlation coefficient between these spatial labels and the pseudotime values inferred based on embeddings from RGAST and other baseline methods. The results demonstrate that RGAST achieves a remarkably high Spearman correlation of 0.958 (Fig. 5d). This indicates that the trajectory inferred by RGAST preserves the topological order of the cortical layers with high fidelity. As shown in the fitted curves, RGAST presents a smooth, monotonic progression across layers. In contrast, other methods exhibit varying degrees of “backtracking” (oscillating pseudotime values) or chaotic mixing between layers, resulting in lower correlation scores.

### RGAST can reveal 3D spatial patterns with multiple ST sections

**Figure 6:**
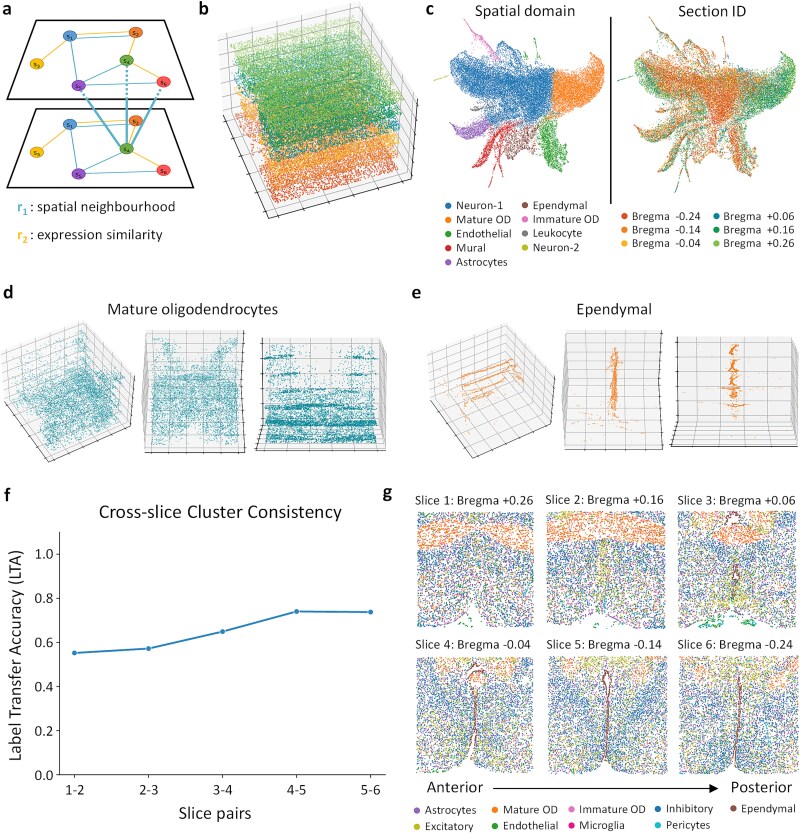
RGAST can reveal 3D spatial patterns with multiple ST sections. (a) Cross-sectional 3D RGAST is constructed by simultaneously considering the 2D relationships within each section and the spatial neighborhoods between adjacent sections. (b) The aligned six mouse hypothalamic preoptic sections profiled using MERFISH. (c) The left panel shows the UMAP visualization of the RGAST embedding colored by manually annotated spatial domains. The right panel shows the UMAP visualization of the RGAST embedding colored by different sections. (d) Visualization of the 3D structure pattern of mature oligodendrocytes from different angles. (e) Visualization of the 3D structure pattern of Ependymal from different angles. (f) Distribution of LTA across all adjacent section pairs. (g) The cell type annotation of each slice from anterior to posterior.

A single slice of ST data can provide a 2D spatial pattern of a specific organ. When multiple consecutive slices are available for the same organ, we can leverage the relationships between these slices to achieve a more precise representation of each spot. In this study, we extended RGAST to handle multiple ST data by simultaneously considering the 2D relationships within each section and the spatial neighborhoods between adjacent sections (Fig. 6a, Materials and Methods). To demonstrate the effectiveness of our approach, we applied RGAST to the mouse hypothalamic preoptic MERFISH data with six consecutive sections (Fig. 6b).

Based on the clustering results in the spatial domain, we manually annotated the cell type of each domain using marker genes (Fig. 6c and Supplementary Fig. S25). The UMAP visualization of RGAST embeddings demonstrates effective integration of spots across different tissue sections (Fig. 6c). This integration performance suggests RGAST’s capability to mitigate technical artifacts, including batch effects. In the 3D spatial domain results detected by RGAST, a clear structure pattern was observed for mature oligodendrocytes and ependymal cells (Fig. 6d and e), which is consistent with previous studies [16]. Additional visualizations of other spatial domains can be found in Supplementary Fig. S26. We further introduced label transfer accuracy (LTA) as a metric to quantify the cross-slice continuity of the inferred 3D domains (Materials and Methods). High accuracy implies that the 3D reconstruction successfully aligns spatially corresponding structures across sections without abrupt disruptions.

As illustrated in Fig. 6f, our method achieves a mean LTA of 0.65, demonstrating an overall continuity across the *Z*-axis. We observed that the LTA values are relatively lower in anterior sections and higher in posterior sections. This observation aligns with the anatomical properties of this specific tissue (e.g. ependymal structure is visually recognizable only after slice 2), where anterior regions exhibit more rapid structural transitions and higher heterogeneity across slices, whereas posterior regions present a more stable and consistent architecture over the *Z*-axis (Fig. 6g). This correspondence confirms that our 3D reconstruction not only achieves good alignment but also faithfully preserves the intrinsic biological variability of the tissue structure.

These results illustrate that RGAST can facilitate analyzing across different tissue sections and accurately capture 3D expression patterns by incorporating spatial information.

## Discussion

In this study, we introduced RGAST, a versatile computational framework designed to address the multifaceted analytical challenges posed by spatial transcriptomics data. We have demonstrated that by leveraging a relational graph attention auto-encoder, RGAST generates *de novo* cell–cell communication networks and powerful latent embeddings that serve as a robust foundation for a wide array of downstream applications. Our results consistently show its superior performance in spatial domain identification across diverse ST platforms, its novel capability in inferring multi-scale cell–cell communication networks, and its enhanced utility in SVG detection, trajectory inference, and 3D tissue reconstruction.

The principal advantage of RGAST lies in its heterogeneous graph structure, which concurrently models both spatial proximity and gene expression similarity. This addresses a critical gap in ST analysis: the trade-off between spatial fidelity and transcriptional resolution. By jointly modeling spatial neighborhoods and expression profiles, RGAST generates low-dimensional embeddings that outperform SOTA clustering tools (e.g. BayesSpace, STAGATE) in delineating tissue architectures. For instance, in the 10× Visium DLPFC dataset, RGAST improved clustering accuracy (ARI = 0.633) by ~10% compared to the next-best method, highlighting its utility in identifying spatially coherent domains. Crucially, we extended the adaptive attention utility to deciphering multi-scale cell–cell communication networks. Our analysis of the mouse hypothalamus successfully reconstructed known neuroglial interactions, including functionally significant long-range signaling pathways such as the Penk-Oprd1 interaction between inhibitory and excitatory neurons, which are often missed by conventional methods. Moreover, RGAST’s embeddings facilitated the detection of SVGs with higher Moran’s I score, revealing biologically meaningful patterns (e.g. cortical layer-specific markers) that were less discernible with other methods. Trajectory analysis suggests that RGAST provides a more accurate representation of cell trajectories compared to other methods. Additionally, the cross-sectional 3D RGAST analysis, though preliminary, provides a foundation for reconstructing volumetric tissue models from serial sections, mitigating batch effects through cross-section alignment.

While numerous computational tools have been developed for ST data analysis, many are specialized for a single task. RGAST is positioned as a unified framework with improved performance across the analytical spectrum. Unlike methods that focus solely on spatial proximity, which may obscure functional relationships, or non-spatial methods like Seurat that ignore tissue context, RGAST provides a holistic representation. This makes it a powerful upstream tool that can be flexibly applied to various biological questions, from deciphering tissue organization to mapping complex intercellular signaling circuits.

In conclusion, RGAST represents a significant advancement in the computational analysis of spatial transcriptomics data. By providing a versatile and powerful framework that excels at a wide range of analytical tasks, RGAST empowers researchers to uncover the complex spatial organization of tissues with higher fidelity. We anticipate that RGAST will become a valuable tool for the scientific community, accelerating discoveries in developmental biology, neuroscience, and oncology.

**Key Points**

- RGAST leverages a relational graph attention auto-encoder to jointly model spatial proximity and gene expression similarity, enabling simultaneous capture of local and global structures in spatial transcriptomics data, and serves as a unified framework eliminating the fragmentation of existing analytical workflows.
- Across diverse ST platforms, RGAST outperforms SOTA methods in spatial domain identification.
- RGAST enables *de novo* single-cell resolution cell–cell communication analysis, reconstructing known neuroglial interactions and achieving better performance than SOTA CCC tools.
- RGAST also excels in other critical downstream tasks: it enhances spatially variable gene detection, enables precise developmental trajectory inference in the human cortex, and robustly reconstructs 3D tissue architectures from serial ST sections.

## Supplementary Material

supplementary_information_revise_bbag298

## Data Availability

All data used in this research can be found in Supplementary Table S1. Our RGAST method is available as a Python package on PyPI at https://pypi.org/project/RGAST, free for academic use, and the source code is openly available from our GitHub repository at https://github.com/GYQ-form/RGAST.
