# Supplementary material for: Empowering multifaceted analysis of spatial transcriptomics data with RGAST
Source: Brief Bioinform. 2026 Jun 16;27(3):bbag298. doi: 10.1093/bib/bbag298 (PMC13271401; doi:10.1093/bib/bbag298)
Supplement: supplementary_information_revise_bbag298 [file supplementary_information_revise_bbag298.docx]

**Supplementary Information**

**Supplementary Tables**

**Table S1. Detailed description of the datasets used in the paper**

| **Datasets** | **Description** | **Source** |
| --- | --- | --- |
| Mouse hypothalamic preoptic | six consecutive slices from a single mouse’s preoptic region of hypothalamus profiled by MERFISH | https://datadryad.org/stash/dataset/doi:10.5061/dryad.8t8s248 |
| Human breast cancer | HDST data obtained from a histological grade 3 HER2+ patient | https://portals.broadinstitute.org/single_cell/study/SCP420 |
| Human DLPFC | 12 human DLPFC sections sampled from three individuals profiled by 10X Visum | http://spatial.libd.org/spatialLIBD/ |
| Mouse olfactory bulb | Data from mouse olfactory bulb tissues profiled by Stereo-seq | https://github.com/JinmiaoChenLab /SEDR_analyses |
| Mouse cortex | seqFISH+ dataset contained mRNAs for 10,000 genes in single cells, with high accuracy and sub-diffraction-limit resolution | https://www.spatialomics.org/SpatialDB/seqfifish_30911168_browse.php |

**Table S2. Curated cell type-specific LR interaction rules used in this study**

| **Sender cell** | **Reciever cell** | **Ligand** | **Receptor** |
| --- | --- | --- | --- |
| Excitatory | Inhibitory | Oxt | Oxtr |
| Inhibitory | Excitatory | Cck | Cckbr |
| Inhibitory | Inhibitory | Tac2 | Tacr3 |
| Excitatory | Inhibitory | Crh | Crhr1 |
| Inhibitory | Excitatory | Penk | Oprd1 |
| Inhibitory | Excitatory | Gal | Galr1 |
| Inhibitory | Excitatory | Nts | Ntsr1 |
| Excitatory | Inhibitory | Trh | Trhr |

**Supplementary Figures**

**Fig S1-S11. clustering results for the remaining 10X Visium DLPFC slices**

151507


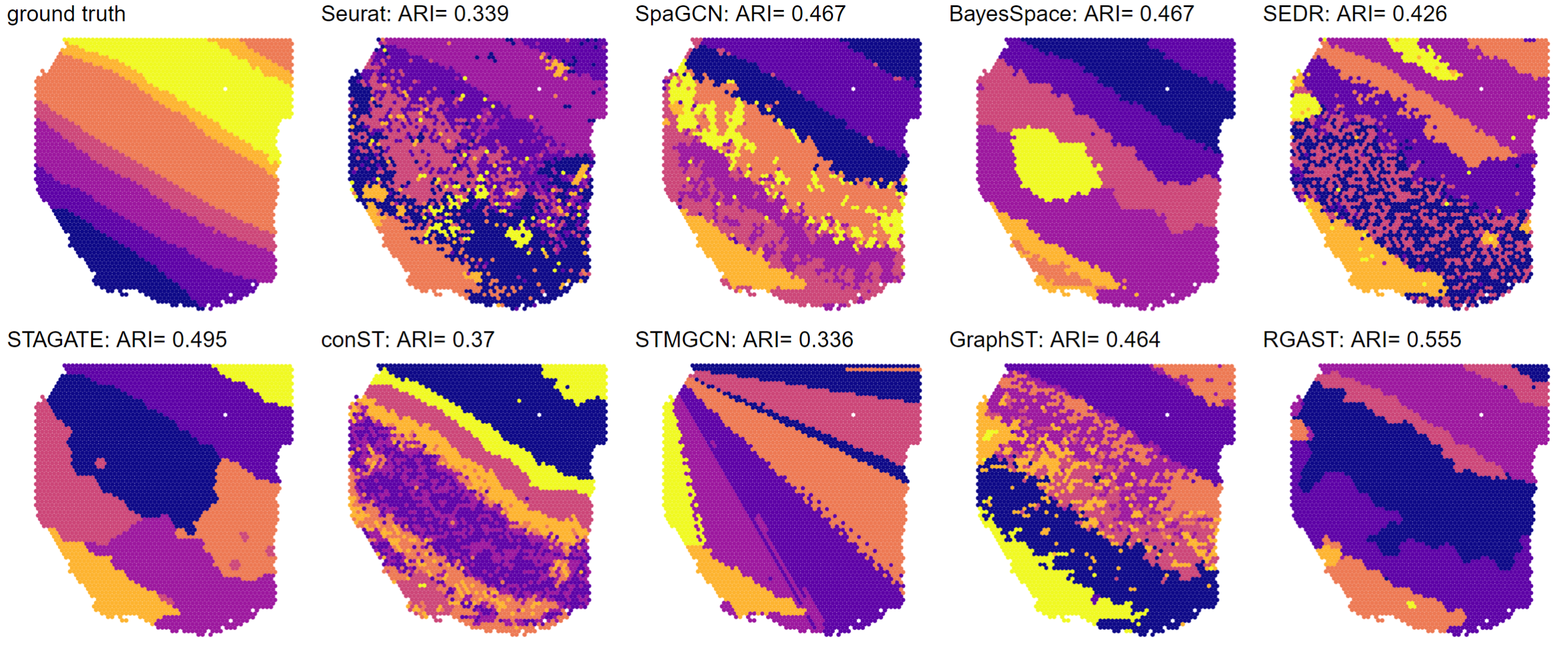


151508


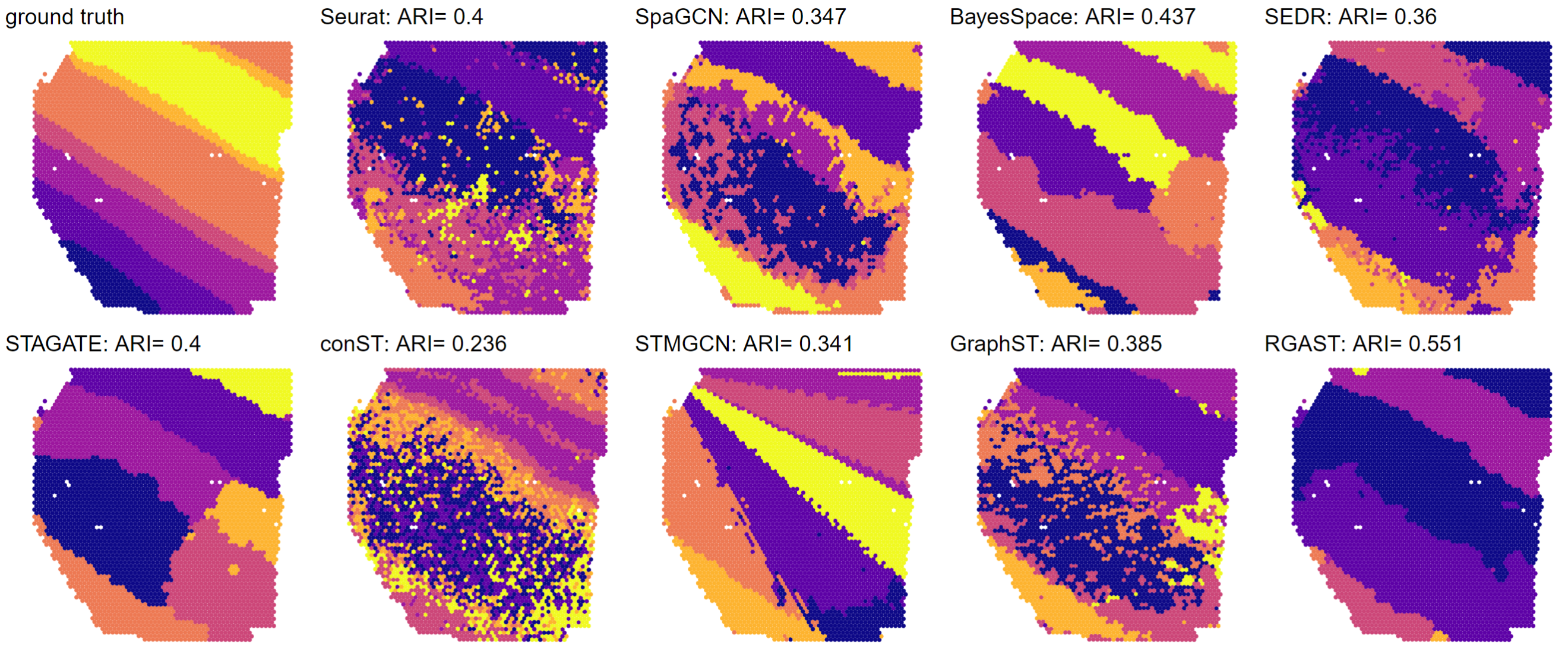


151509


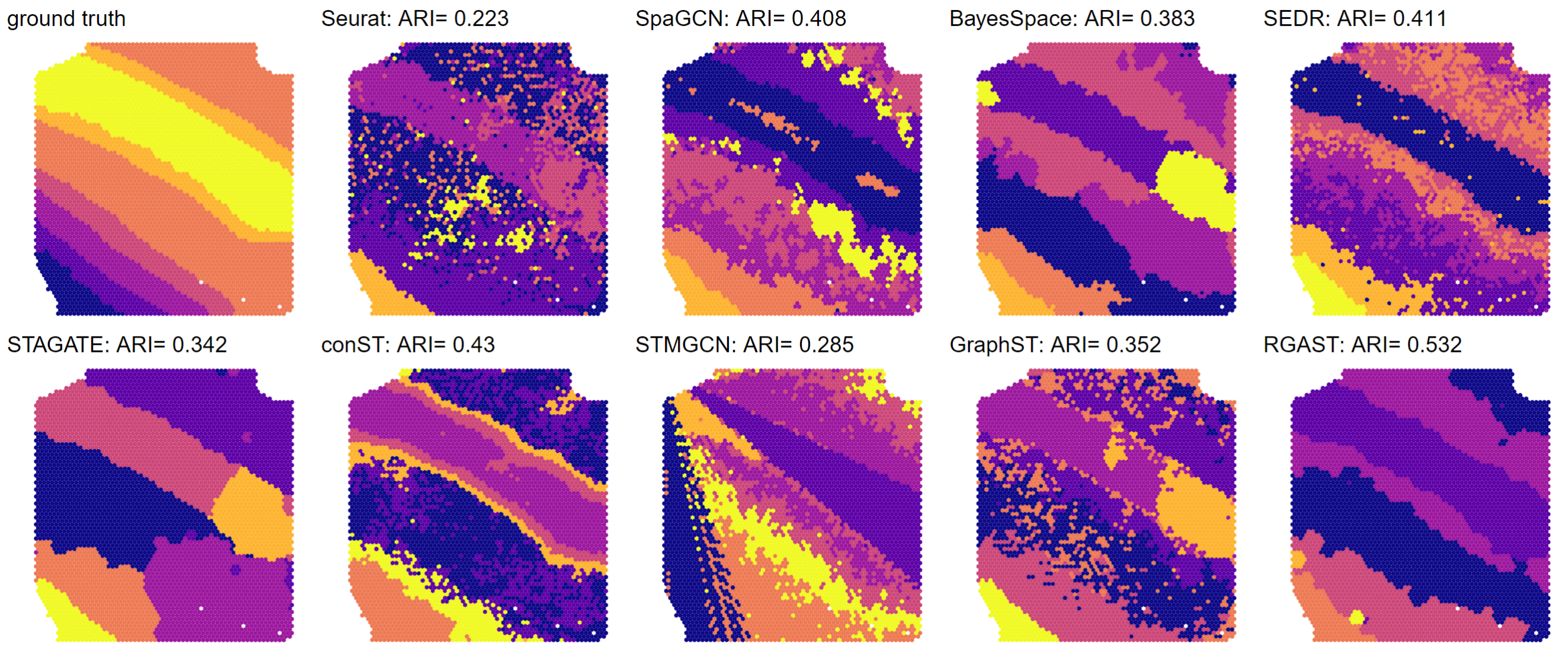


151510


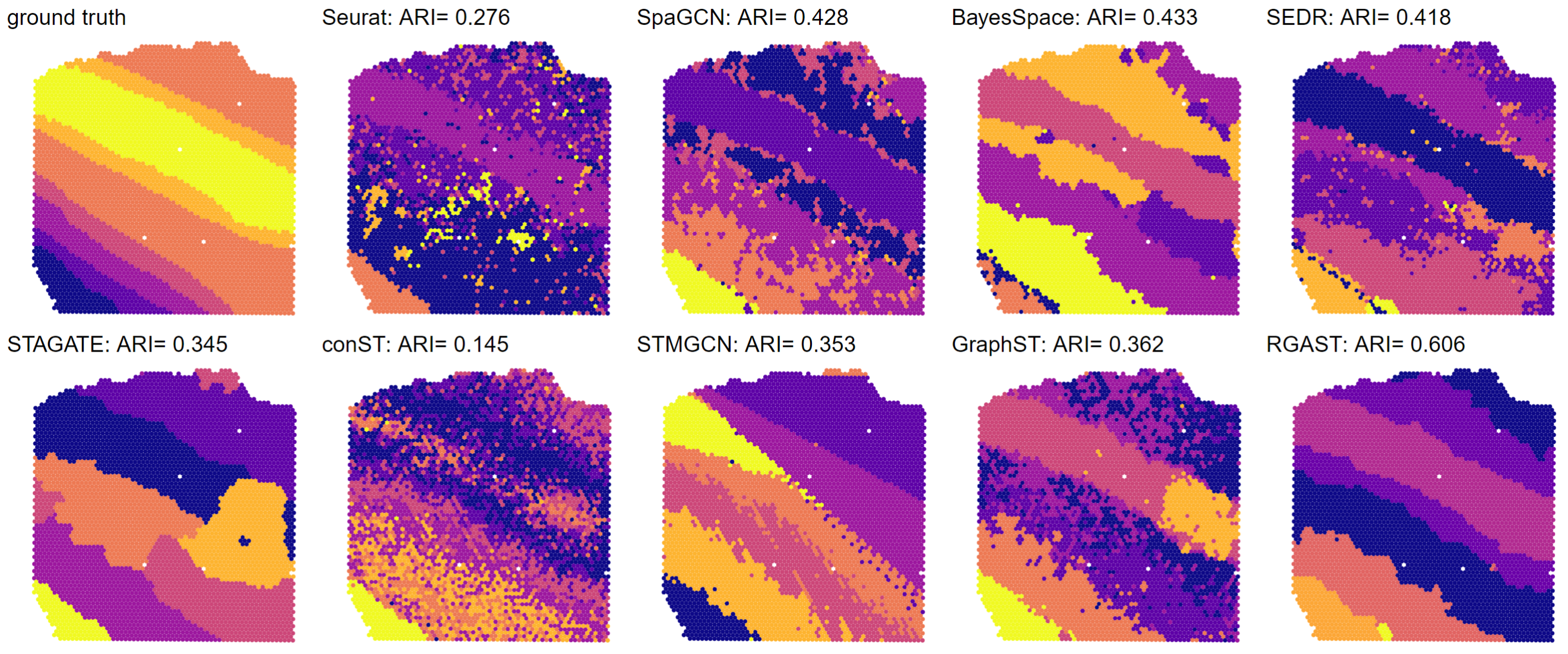


151669


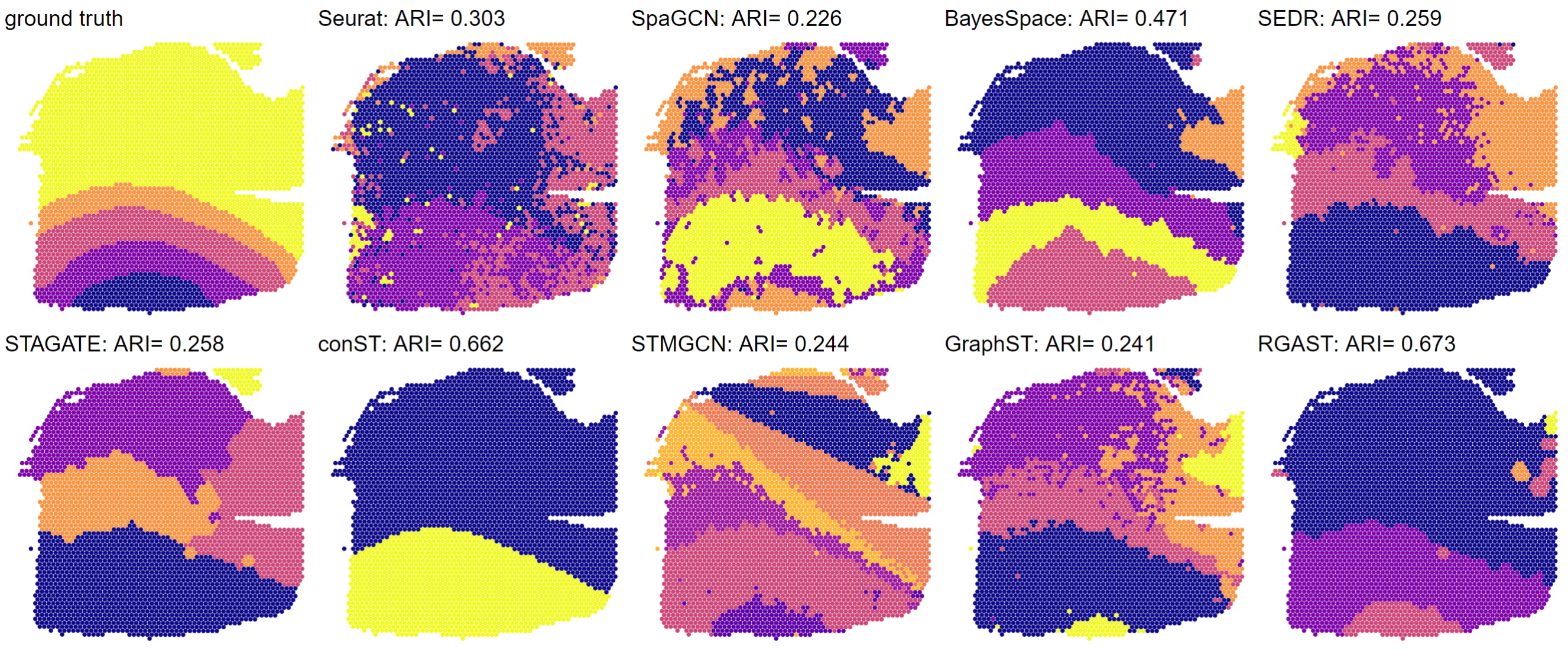


151670


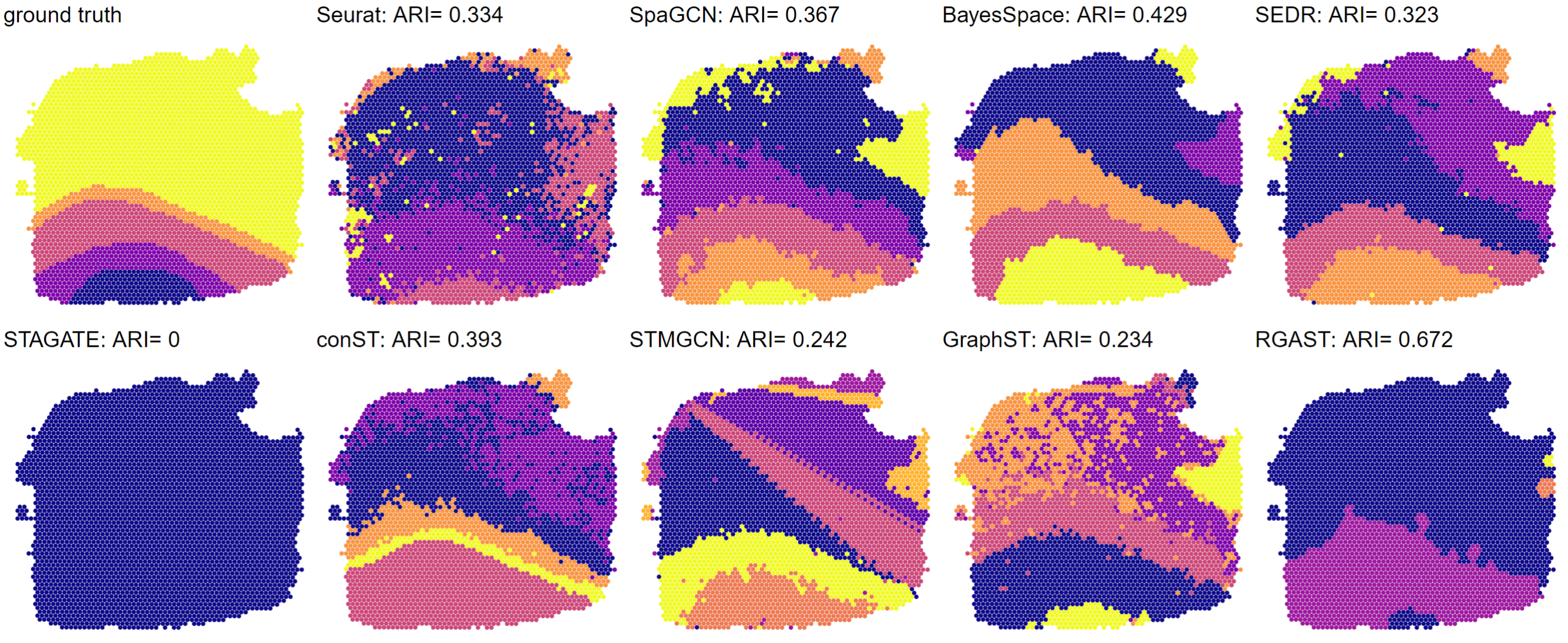


151671


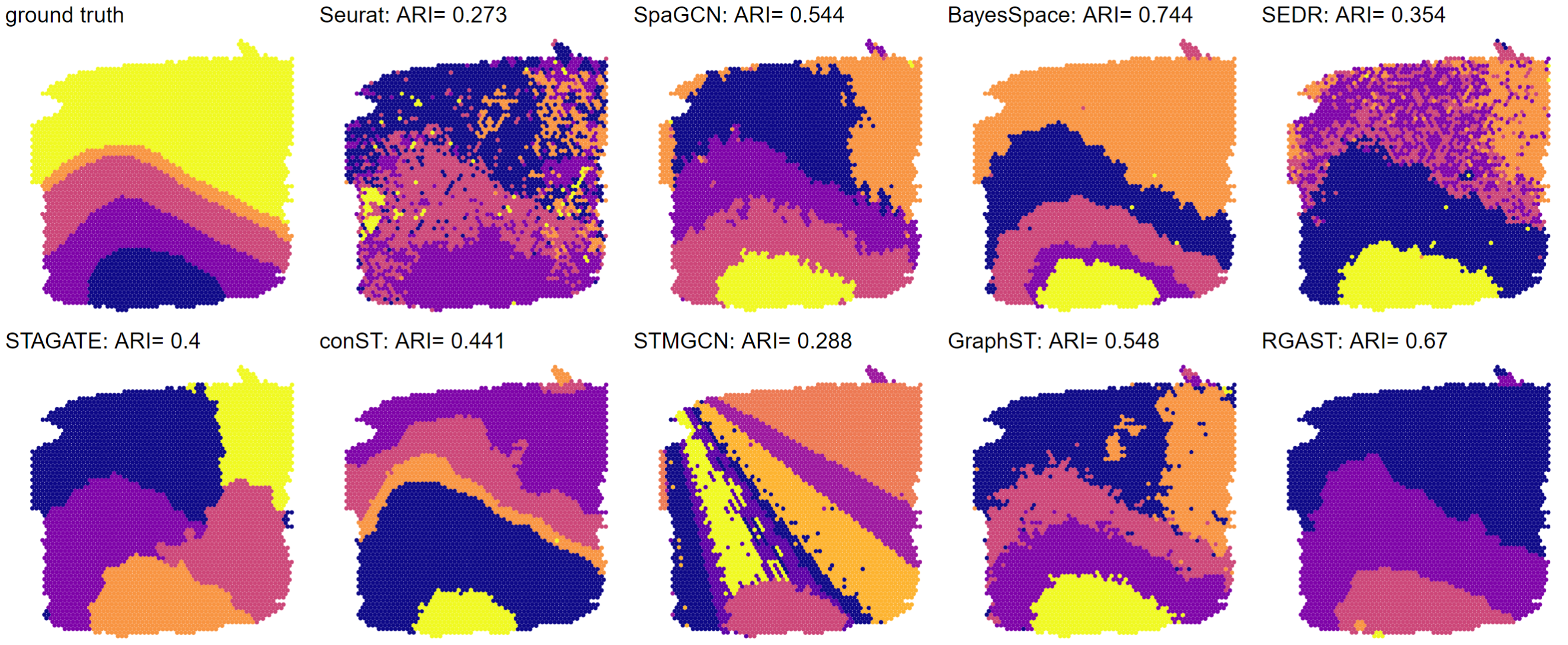


151672


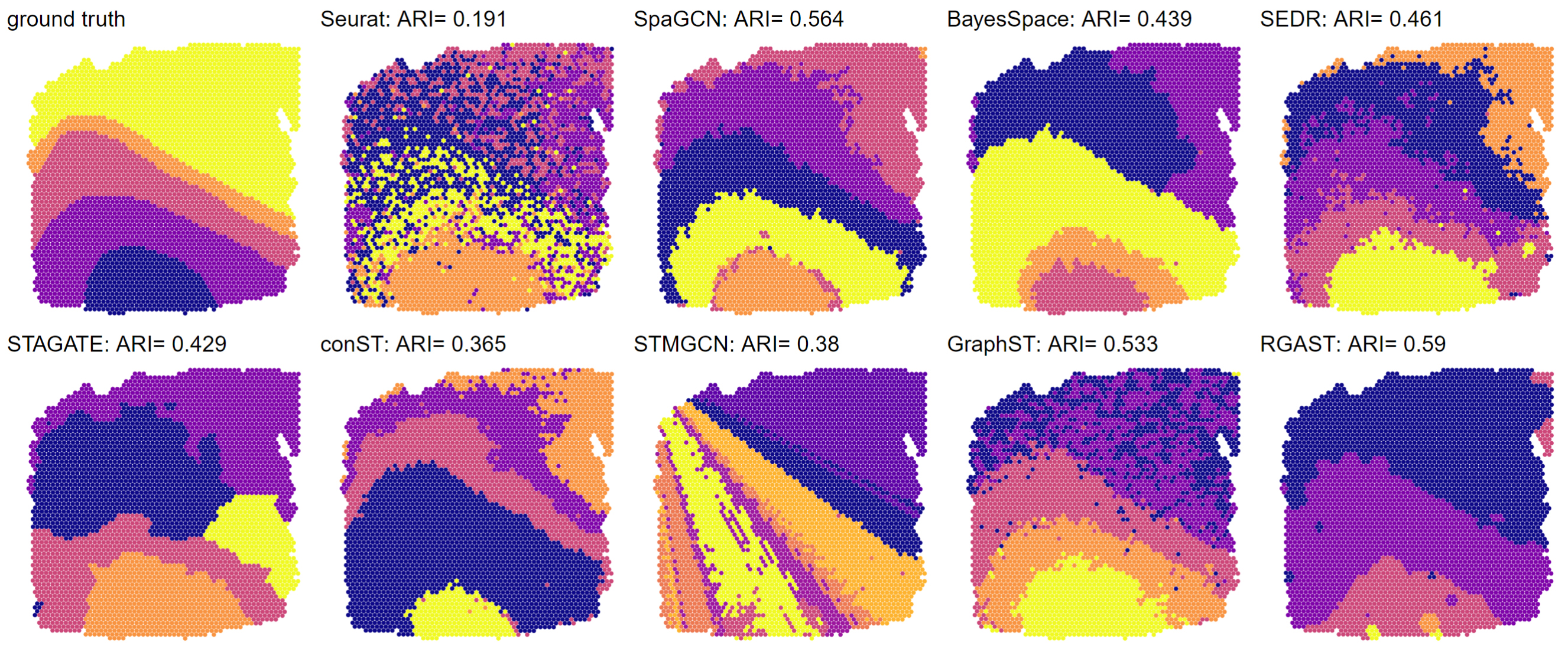


151673


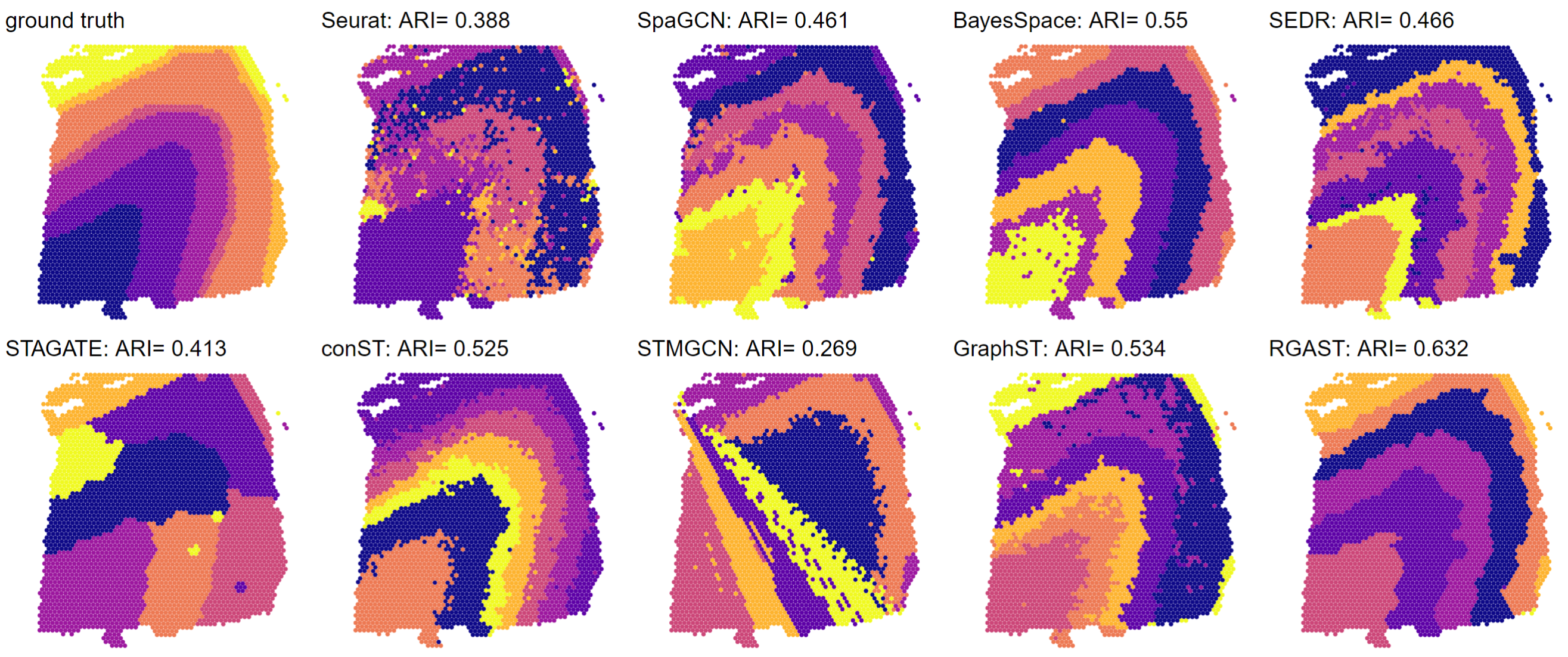


151674


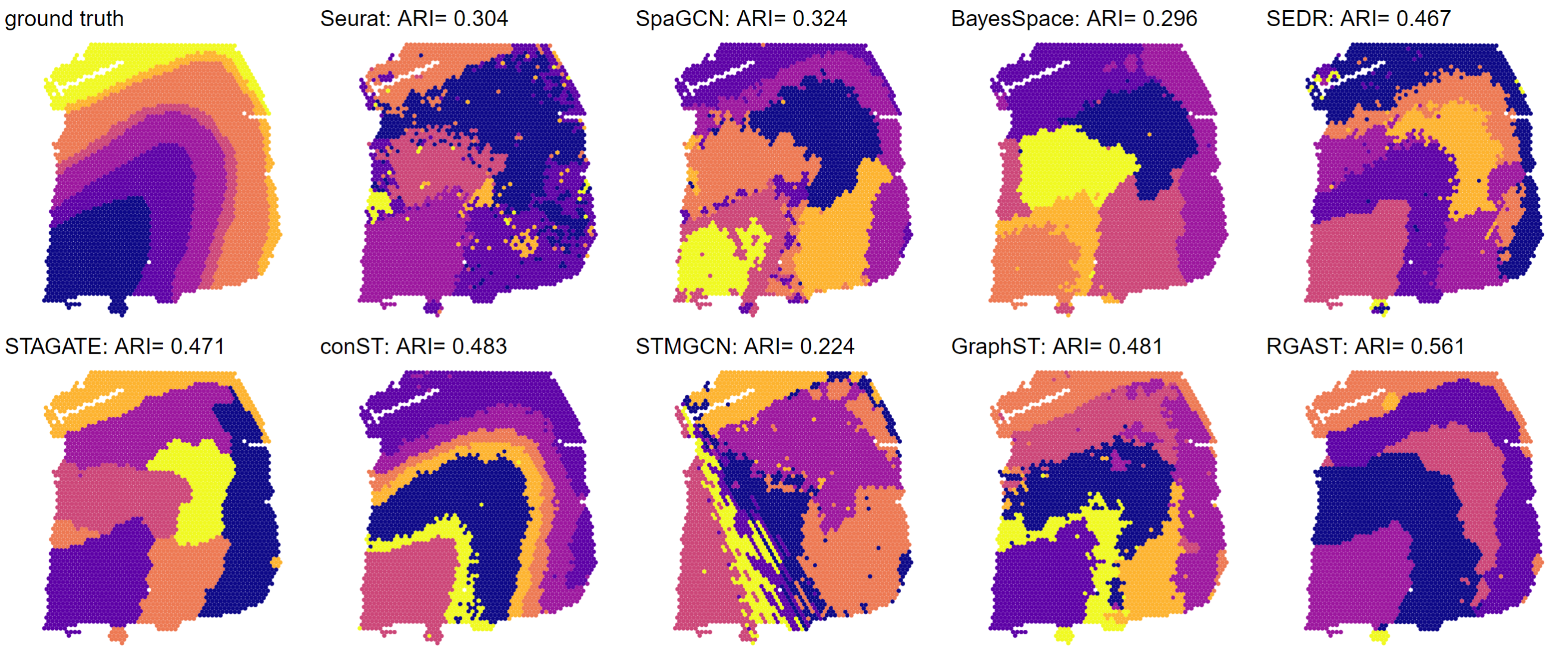


151676


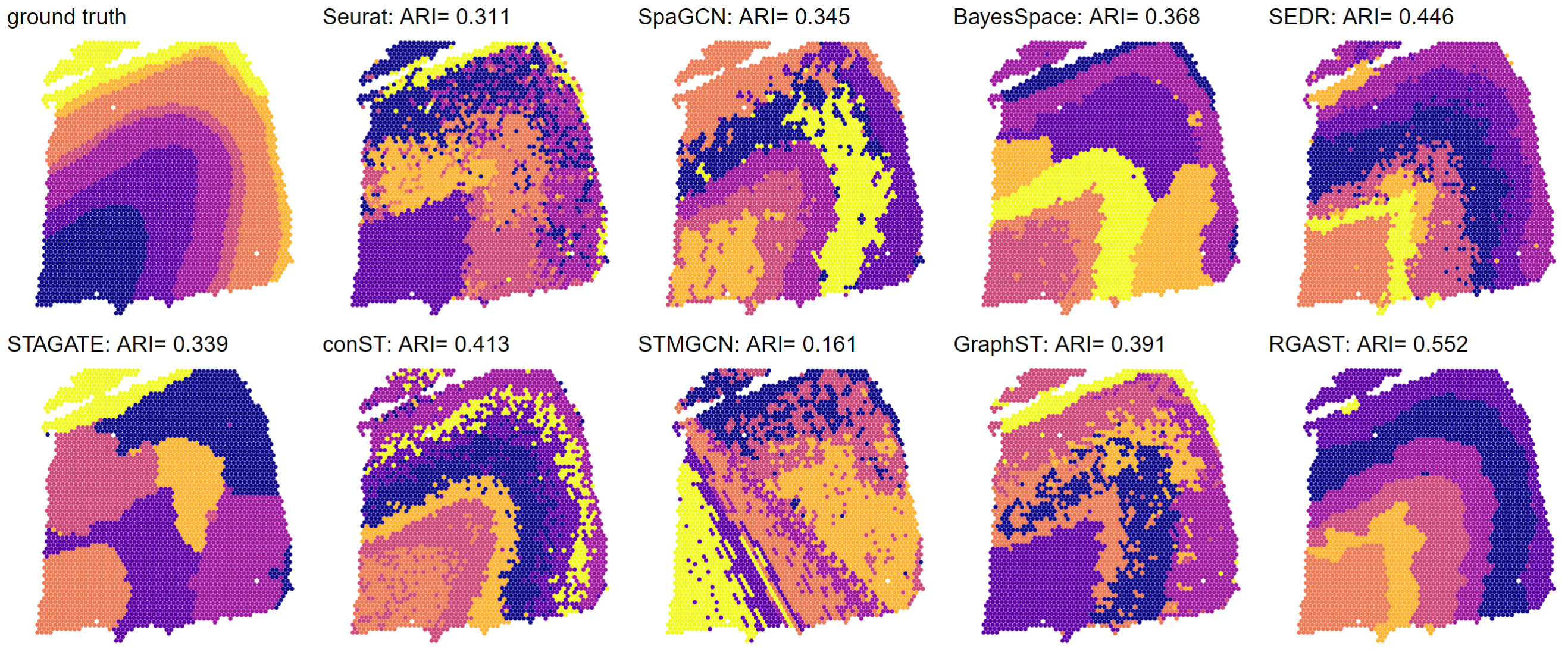


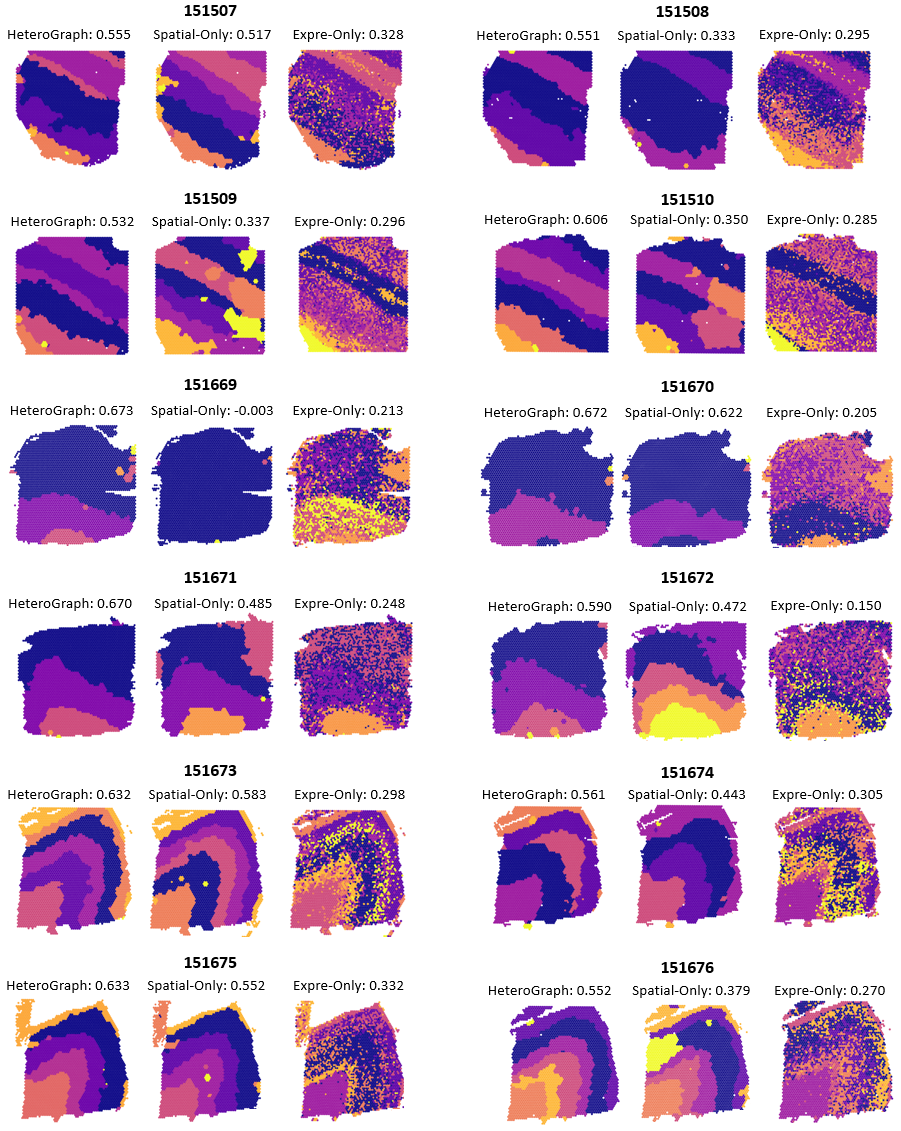


**Fig S12. Ablation study on the contribution of spatial and expression edges in the heterogeneous graph.**


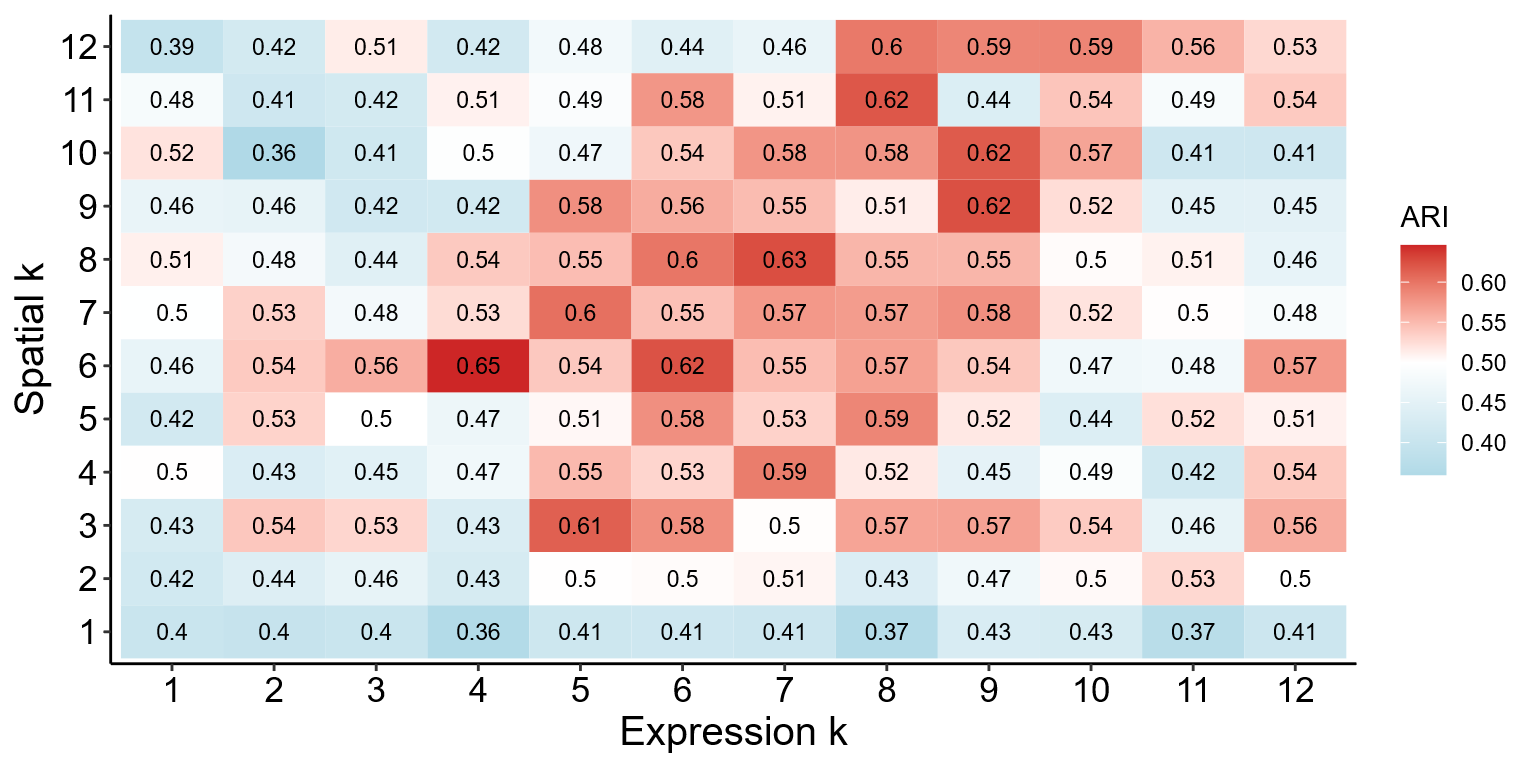


**Fig S13. Sensitivity analysis of graph construction hyperparameters.** Heatmap showing the mean clustering performance (ARI) of RGAST on 12 DLPFC sections under varying graph topologies. The x-axis represents the number of neighbors for the gene expression graph ($k_{e}$), and the y-axis represents the number of neighbors for the spatial graph ($k_{s}$), both ranging from 1 to 12.


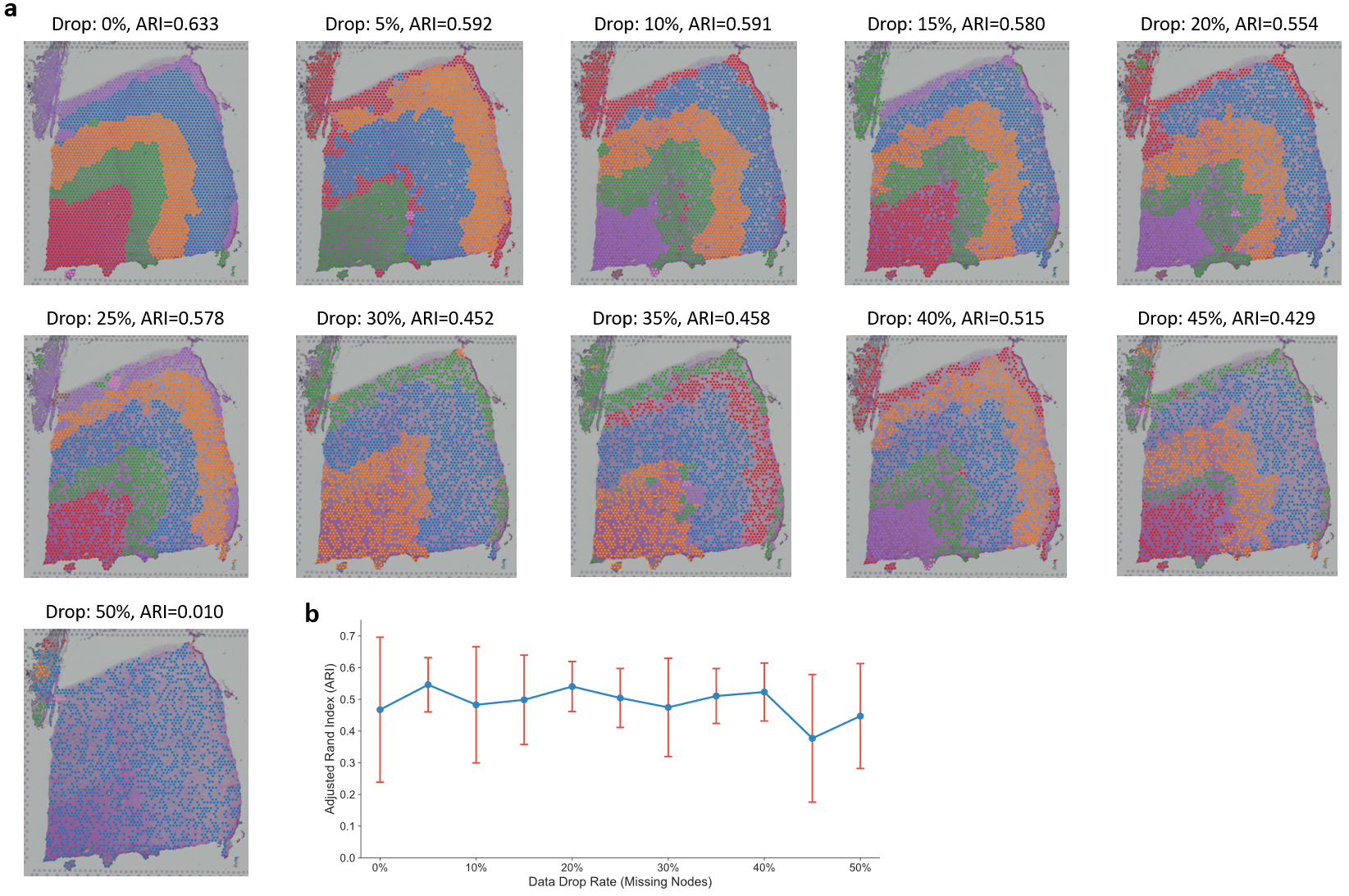


**Fig S14. Robustness analysis of RGAST against data loss and structural noise. a,** Visualization of spatial clustering results on DLPFC slice 151675 under varying data drop rates. **b,** Line plot summarizing the clustering performance (ARI) across all 12 DLPFC slices under different data drop rates. The blue line represents the mean ARI, and the error bars indicate the standard deviation.

**
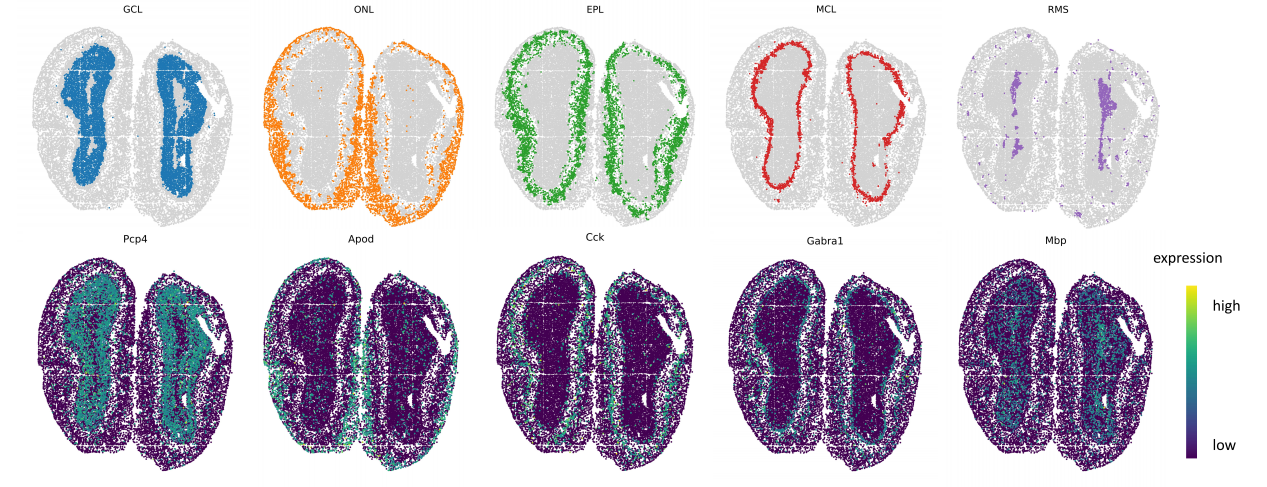
**

**Fig S15. Spatial domains and corresponding marker genes identified by RGAST in Stereo-seq mouse olfactory bulb tissue**

**Fig S16-S20. clustering results for the remaining MERFISH mouse hypothalamic preoptic region slices**

Bergman 0.16


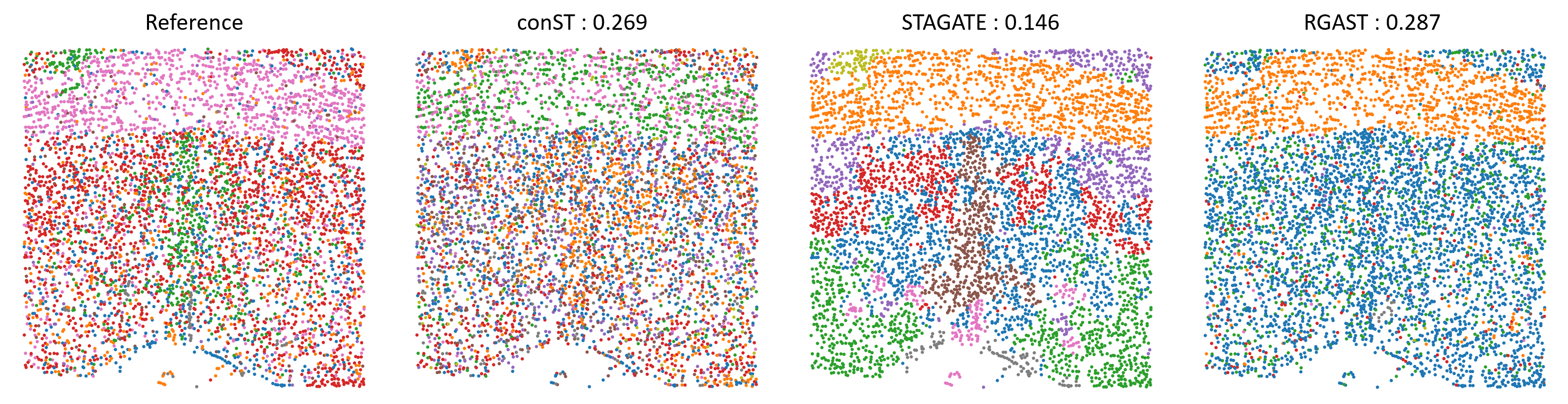


Bergman 0.06


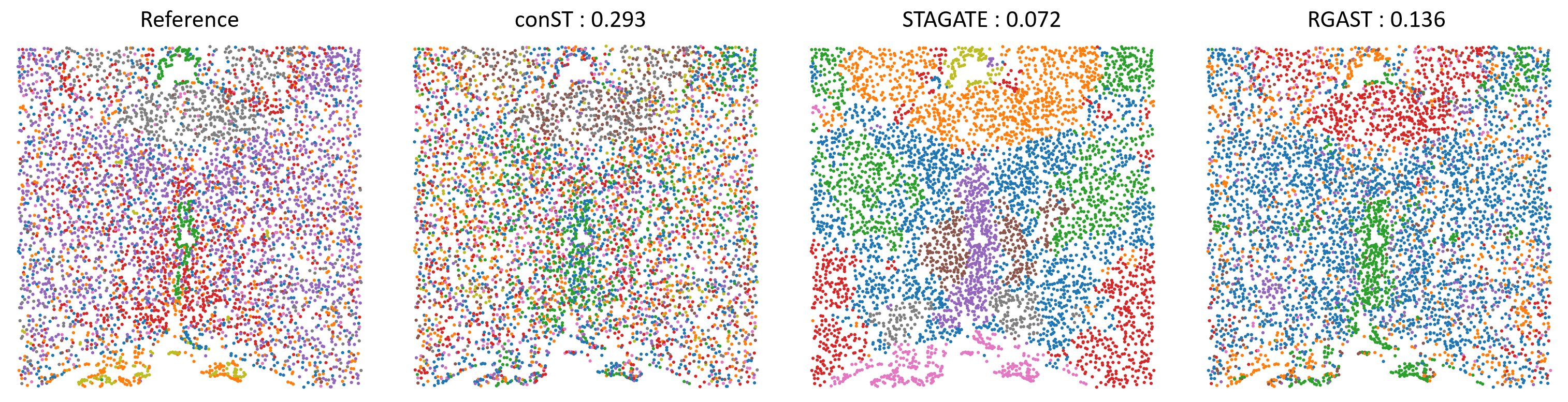


Bergman -0.04


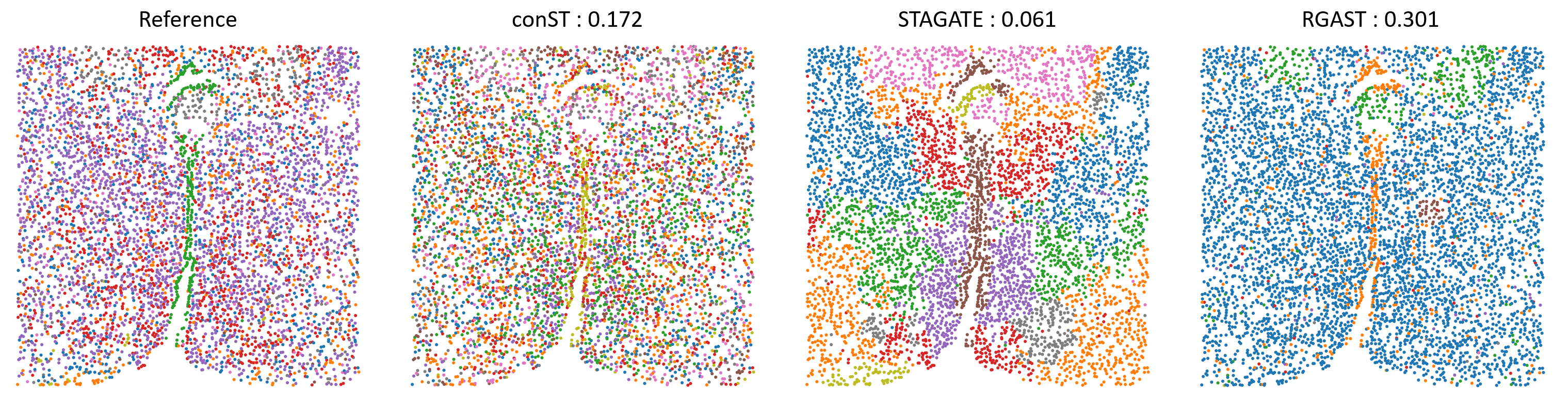


Bergman -0.14


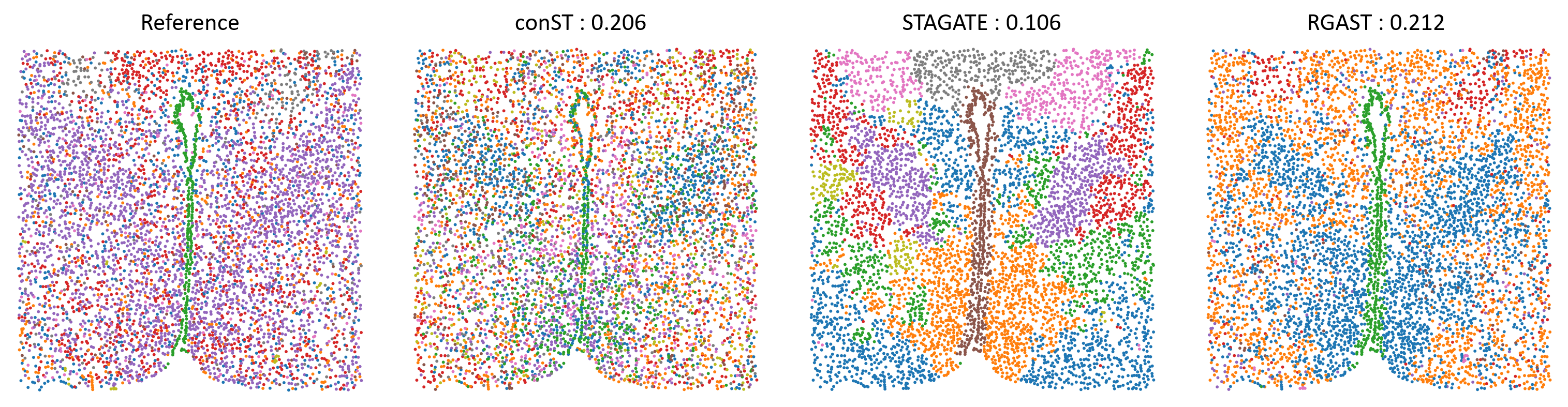


Bergman -0.24


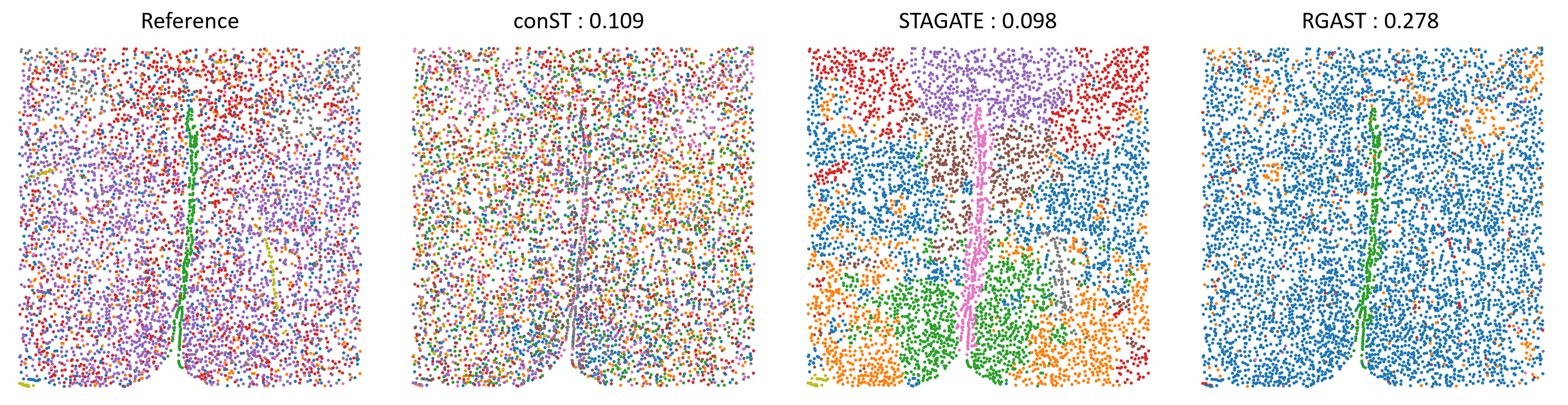


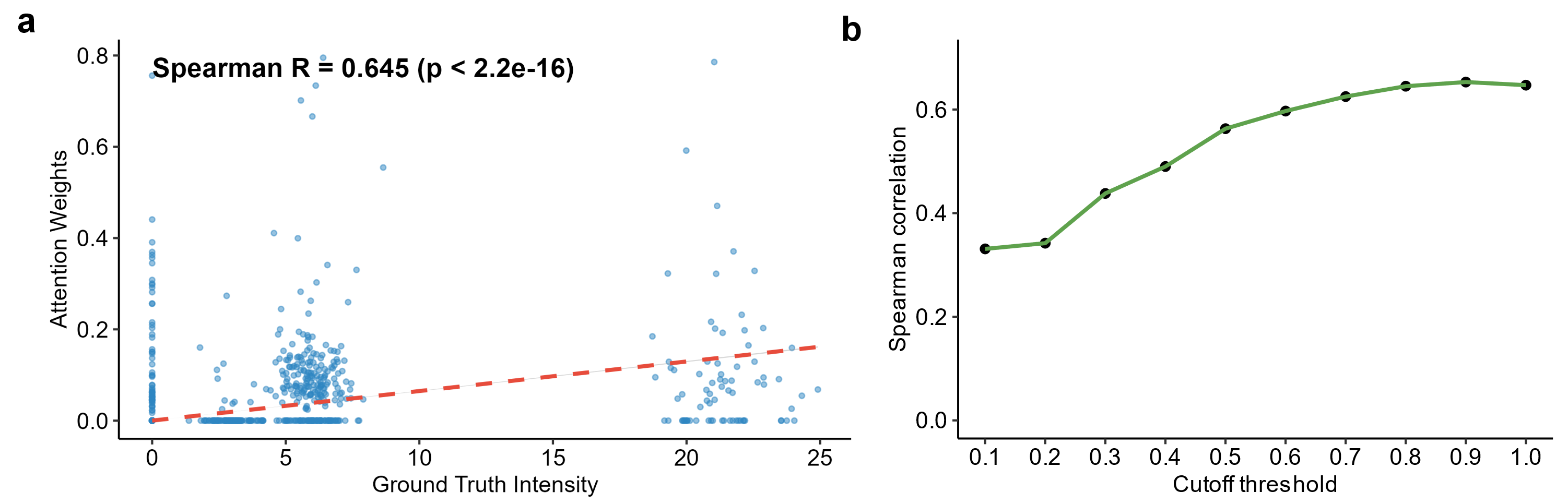
**Fig. S21. | Rationale of attention weights from RGAST as CCC proxies.** **a,** Scatter plot of the learned attention weights from RGAST model versus ground truth interaction intensity between cell pairs. P-value is calculated by large-sample approximate t-test. Dashed red line is the fitted linear regression result. **b,** Sensitivity analysis of the impact of varying attention weight cutoffs (ranging from 0.1 to 1.0 with 0.1 increments) on the strength of the correlation.

**Fig. S22 | Multi-scale CCC analysis in the mouse hypothalamic preoptic region.** **a,** Overall cell type level CCC analysis result on the Bregma +0.26 slice. The up-headed arrow indicates highest communication score, while down-headed arrow indicates lowest communication score. **b,** Short-range single-cell resolution communication comprising astrocyte. **c,** Short-range single-cell resolution communication comprising oligodendrocyte.


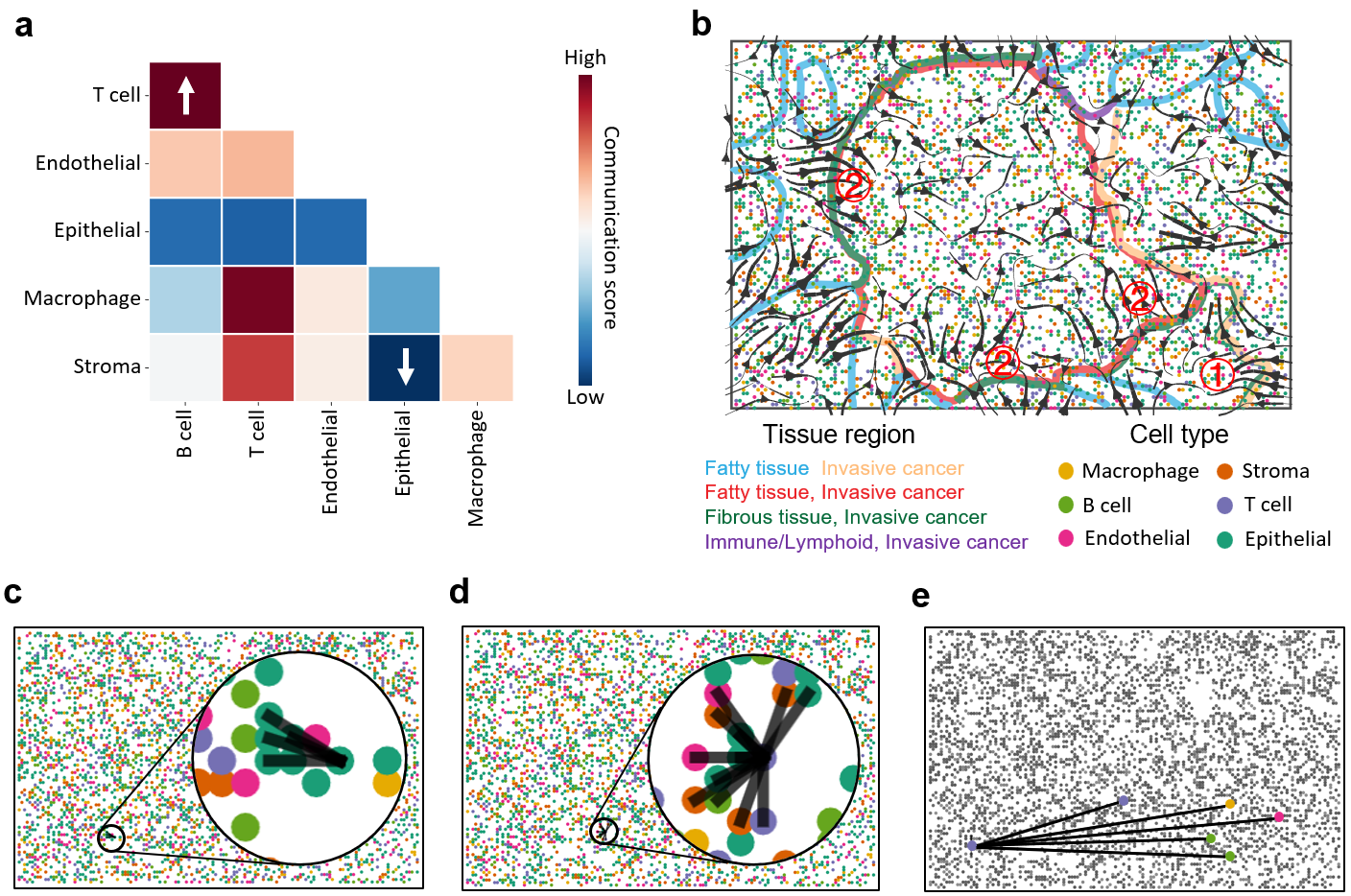


**Fig. S23 | Multi-scale CCC analysis in the HDST breast cancer data.** **a,** Cell type level CCC analysis result. The up-headed arrow indicates highest communication score, while down-headed arrow indicates lowest communication score. **b,** Niche level communication flow plot. The cell type annotation and tissue region segmentation are adopted from Vickovic et al. and are colored identically to the legend. ① indicates the flow from invasive cancer area to fibrous tissue. ② indicates flow from fibrous tissue to fatty tissue-enriched tumor regions. **c,** Short-range single-cell resolution communication of an epithelial cell. **d,** Short-range single-cell resolution communication of a T cell. **e,** Long-range single-cell resolution communication between a T cell and other cells.


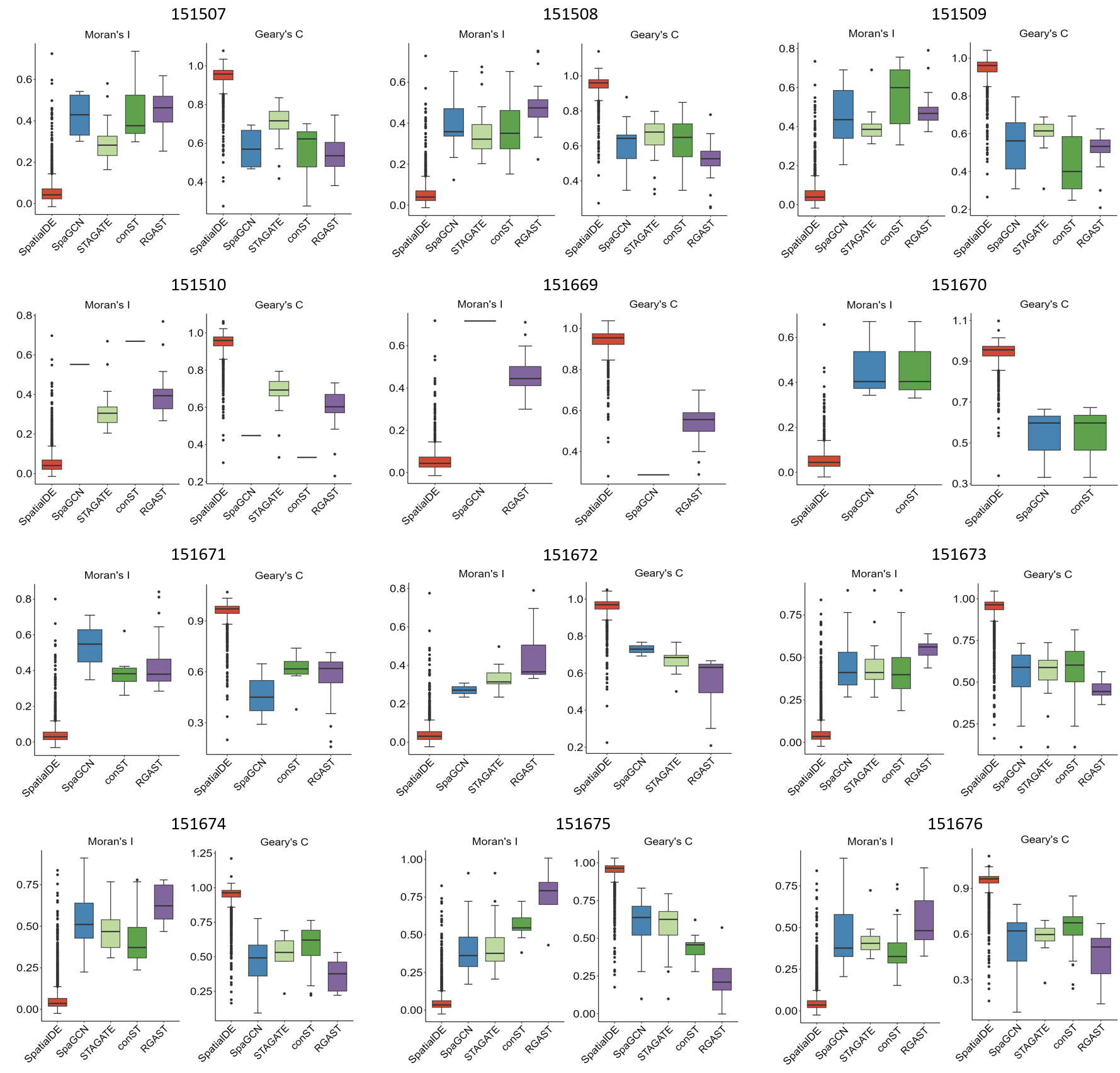


**Fig. S24 | Spatial autocorrelation of SVGs identified by different methods across 12 DLPFC slices.** Box plots summarizing the Moran’s I and Geary’s C for SVGs identified by different methods, including the embedding-independent method SpatialDE, and 4 embedding-dependent methods across all 12 DLPFC sections. Methods with no identified SVG are not shown in specific comparison.


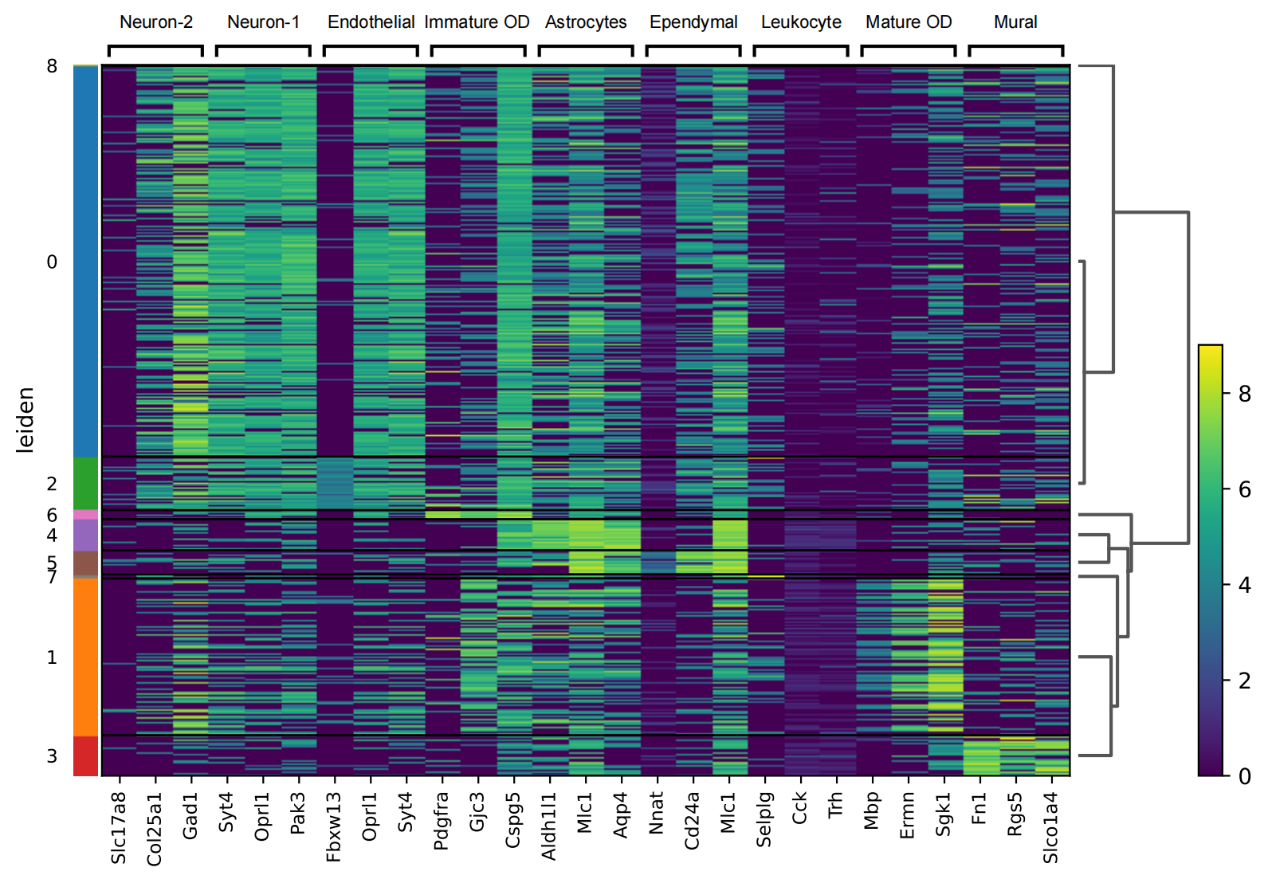


**Fig S25. Heatmap of gene expression in the merged MERFISH data**

**
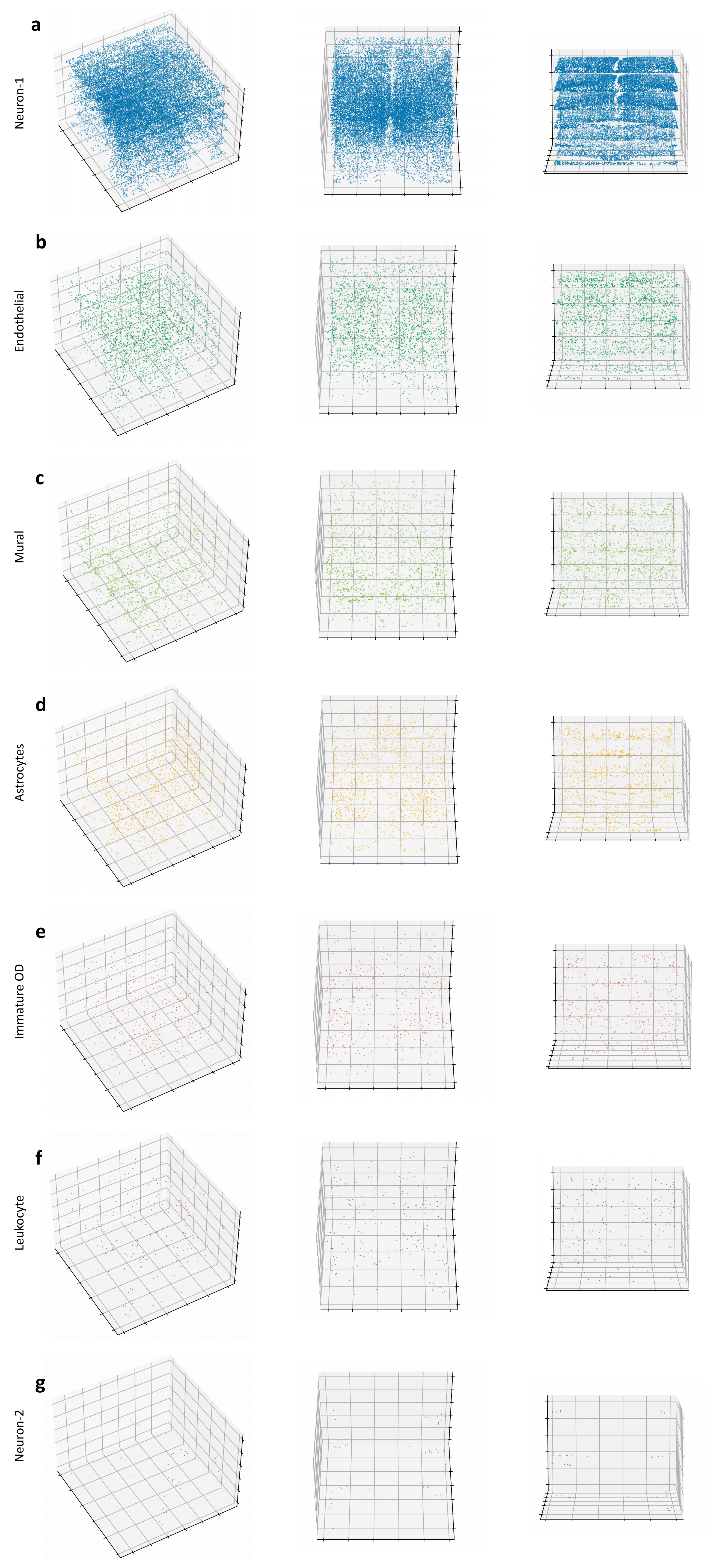
**

**
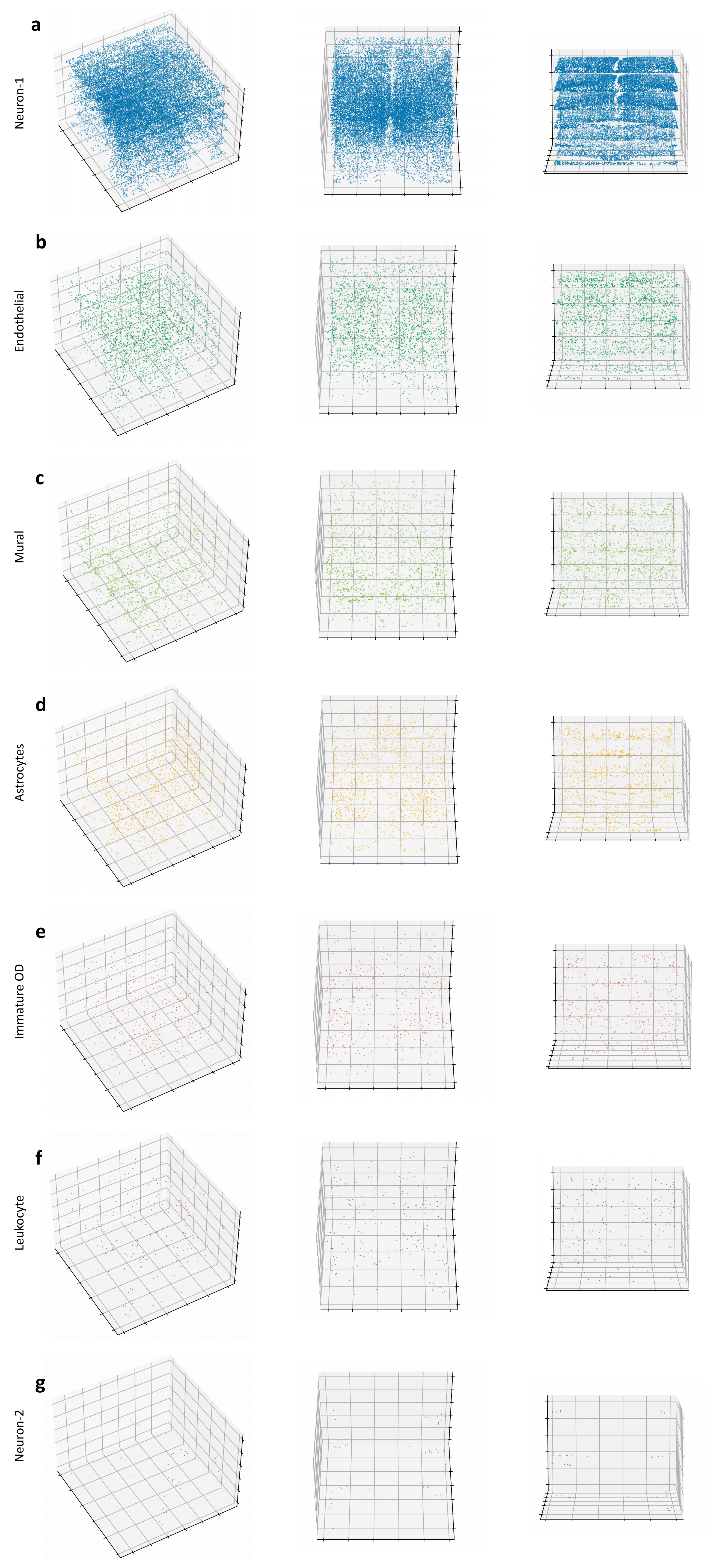
**

**Fig S26. 3D Spatial domain of MERFISH data identified by RGAST**
